# Mitophagy protects **β** cells from inflammatory damage in diabetes

**DOI:** 10.1172/jci.insight.141138

**Published:** 2020-12-17

**Authors:** Vaibhav Sidarala, Gemma L. Pearson, Vishal S. Parekh, Benjamin Thompson, Lisa Christen, Morgan A. Gingerich, Jie Zhu, Tracy Stromer, Jianhua Ren, Emma C. Reck, Biaoxin Chai, John A. Corbett, Thomas Mandrup-Poulsen, Leslie S. Satin, Scott A. Soleimanpour

**Affiliations:** 1 Division of Metabolism, Endocrinology and Diabetes and Department of Internal Medicine, and; 2 Department of Pharmacology, University of Michigan Medical School, Ann Arbor, Michigan, USA.; 3 Department of Biomedical Sciences, University of Copenhagen, Copenhagen, Denmark.; 4 Program in Biological Sciences, University of Michigan Medical School, Ann Arbor, Michigan, USA.; 5 Department of Biochemistry, Medical College of Wisconsin, Milwaukee, Wisconsin, USA.; 6 VA Ann Arbor Healthcare System, Ann Arbor, Michigan, USA.

**Keywords:** Endocrinology, Apoptosis survival pathways, Diabetes, Mitochondria

## Abstract

Inflammatory damage contributes to β cell failure in type 1 and 2 diabetes (T1D and T2D, respectively). Mitochondria are damaged by inflammatory signaling in β cells, resulting in impaired bioenergetics and initiation of proapoptotic machinery. Hence, the identification of protective responses to inflammation could lead to new therapeutic targets. Here, we report that mitophagy serves as a protective response to inflammatory stress in both human and rodent β cells. Utilizing in vivo mitophagy reporters, we observed that diabetogenic proinflammatory cytokines induced mitophagy in response to nitrosative/oxidative mitochondrial damage. Mitophagy-deficient β cells were sensitized to inflammatory stress, leading to the accumulation of fragmented dysfunctional mitochondria, increased β cell death, and hyperglycemia. Overexpression of *CLEC16A,* a T1D gene and mitophagy regulator whose expression in islets is protective against T1D, ameliorated cytokine-induced human β cell apoptosis. Thus, mitophagy promotes β cell survival and prevents diabetes by countering inflammatory injury. Targeting this pathway has the potential to prevent β cell failure in diabetes and may be beneficial in other inflammatory conditions.

## Graphical abstract

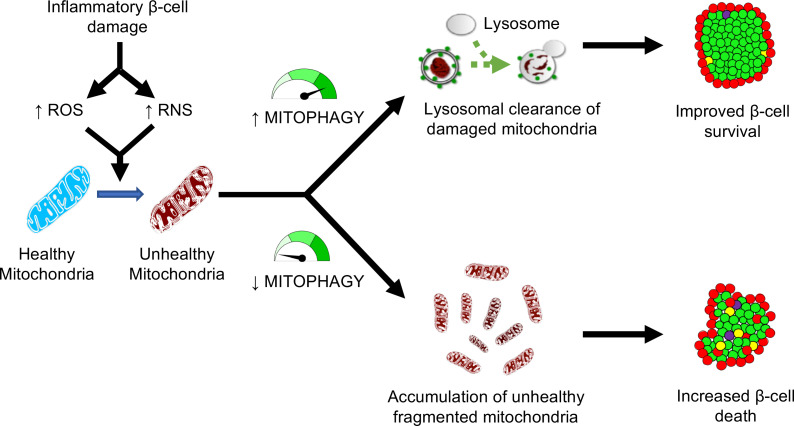

## Introduction

Inflammatory stress plays a crucial role in the pathogenesis of several metabolic diseases, including diabetes (1–3). All forms of diabetes share a common etiology of insufficient pancreatic β cell mass or function to meet peripheral insulin demand, and inflammatory injury is commonly associated with β cell dysfunction (1, 4, 5). Although the precise molecular mechanisms are unclear, the excess generation of free radicals, including nitric oxide (NO) and/or ROS, likely contribute to β cell inflammatory damage (6). Mitochondria are adversely affected by inflammatory signaling in β cells, which can result in impaired bioenergetics, blunted glucose-stimulated insulin secretion, and activation of apoptosis (7). Therefore, strategies to block inflammation and/or preserve mitochondrial function are of great interest as potential diabetes therapies.

Mitochondria exist in dynamic networks that undergo continuous remodeling and respond to both internal and external environmental cues. Mitophagy is an essential quality-control mechanism that selectively eliminates damaged mitochondria to maintain healthy mitochondrial networks (8–10). The type 1 diabetes (T1D) susceptibility gene *CLEC16A* encodes an E3 ubiquitin ligase that controls mitophagic flux in β cells (11–13), indicating a critical role for mitophagy in maintaining β cell function. Indeed, diabetogenic intronic polymorphisms in the *CLEC16A* locus that reduce human islet CLEC16A mRNA expression are associated with impaired β cell function and glucose control in humans (13, 14). Although mitophagy maintains the metabolic function needed for glucose-stimulated insulin release, it has not been shown to affect β cell survival (11, 13, 15). Furthermore, whether mitophagy (or Clec16a) protects β cells from inflammatory attack is unknown.

Here, we elucidate a key protective role for mitophagy in the response to inflammatory stress in β cells. Utilizing in vivo mitochondrial biosensors and biochemical/genetic approaches, we show that proinflammatory cytokines, which model the inflammation that occurs during diabetes pathogenesis, induce mitophagy in both human and rodent β cells. Cytokine-induced free radicals function as upstream inflammatory signals to activate β cell mitophagy, and the impairment of Clec16a-mediated mitophagy exacerbates hyperglycemia and β cell apoptosis following inflammatory stimuli. Lastly, we demonstrate that adenoviral overexpression of CLEC16A protects human β cells against cytokine-mediated demise, illustrating the feasibility of therapeutically targeting this process.

## Results

### Proinflammatory cytokines induce mitochondrial damage and activate β cell mitophagy.

**Figure 1:**
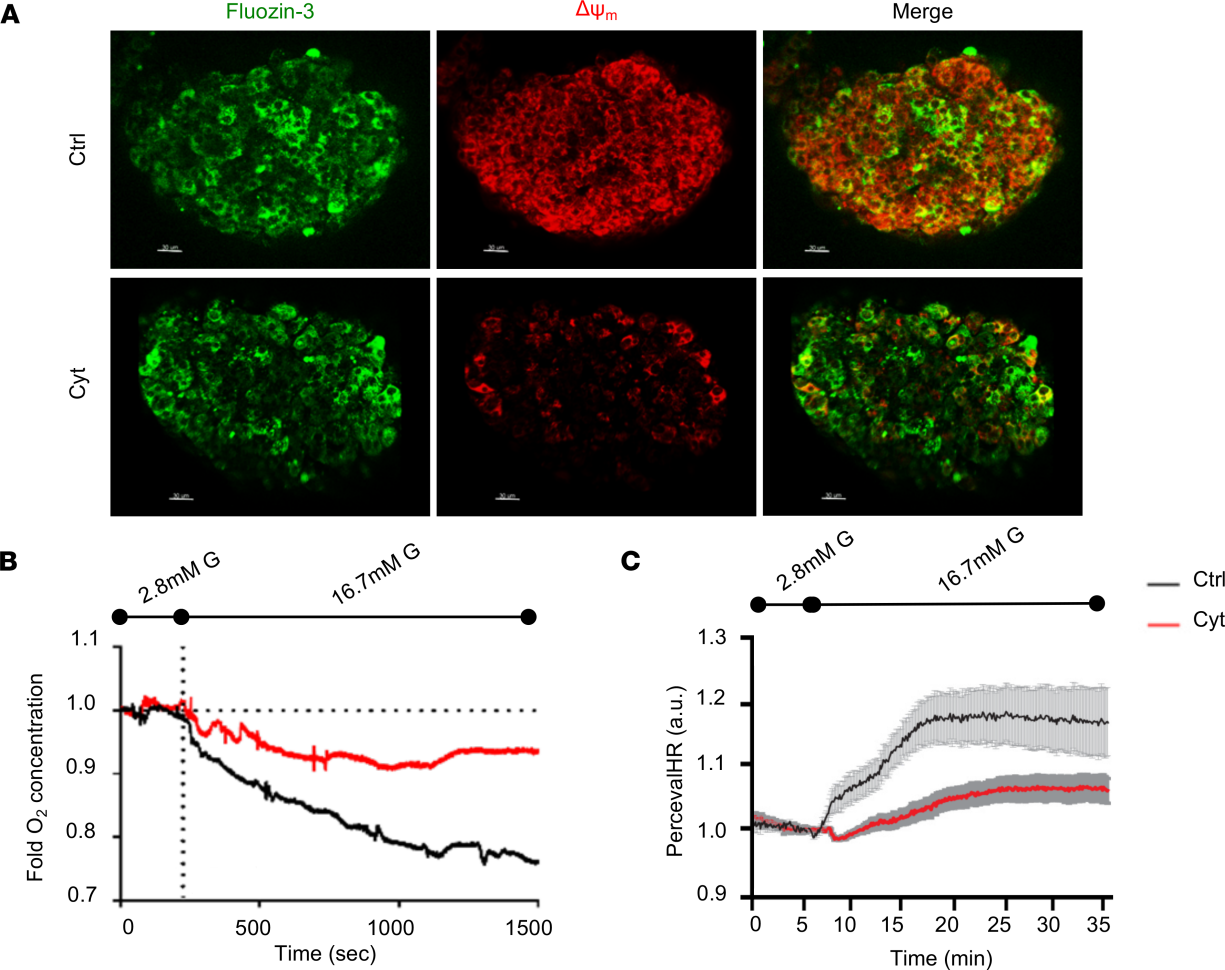
Proinflammatory cytokines impair mitochondrial bioenergetics in human islets. (**A**) Laser scanning confocal microscopy of live human islets at 60× magnification stained with Fluozin-3 (β cells/Zn granules) and TBMS-306 (Δψ_m_) following a 24-hour treatment with control (Ctrl; PBS) or cytokines (Cyt; 75 U/mL IL-1β, 750 U/mL TNF-α, and 750 U/mL IFN-γ). Scale bars: 30 µm. (**B**) O_2_ consumption measured by O_2_ microsensor in Ctrl- and Cyt-treated human islets (*P* < 0.05 by ANOVA). (**C**) ATP/ADP ratios measured by PercevalHR fluorescence in Ctrl- and Cyt-treated human islets (*P* < 0.05 by ANOVA). *n* = 3–6 independent human islet donors/group for all measurements.

Optimal mitochondrial function is central to β cell responses to glucose or other nutrient stimuli. We hypothesized that proinflammatory cytokines induce mitochondrial dysfunction, and β cells then activate mitophagy to eliminate dysfunctional mitochondria. To this end, we first examined the effects of proinflammatory cytokines (combination of IL-1β, TNF-α, and IFN-γ) on mitochondrial function in primary human islets. Mitophagy is initiated following a loss of mitochondrial membrane potential (Δψ_m_) and resultant respiratory dysfunction (13, 16). Utilizing live-cell confocal microscopy, we observed that cytokine exposure dissipated Δψ_m_ primarily in β cells, which were detected by the cell permeable Zn^2+^ dye Fluozin-3 (Figure 1A and ref. 17). Moreover, cytokine exposure reduced both oxygen consumption (Figure 1B) and ATP/ADP ratio (Figure 1C) of human islets in response to glucose stimulation. Glucose-induced increases in the ATP/ADP ratio are necessary for closure of ATP-sensitive potassium (K_ATP_) channels to produce β cell membrane depolarization, and indeed, patch clamping confirmed that cytokine exposure reduced glucose-stimulated membrane depolarization (Supplemental Figure 1A; supplemental material available online with this article; https://doi.org/10.1172/jci.insight.141138DS1). However, β cell depolarization was still seen in response to the sulfonylurea tolbutamide, which closes K_ATP_ channels independently of glucose metabolism, suggesting that the effects of cytokines are metabolic, and thus occur upstream of the K_ATP_ channel (Supplemental Figure 1B). Together, these studies confirm that proinflammatory cytokines induce mitochondrial dysfunction in human β cells.

**Figure 2:**
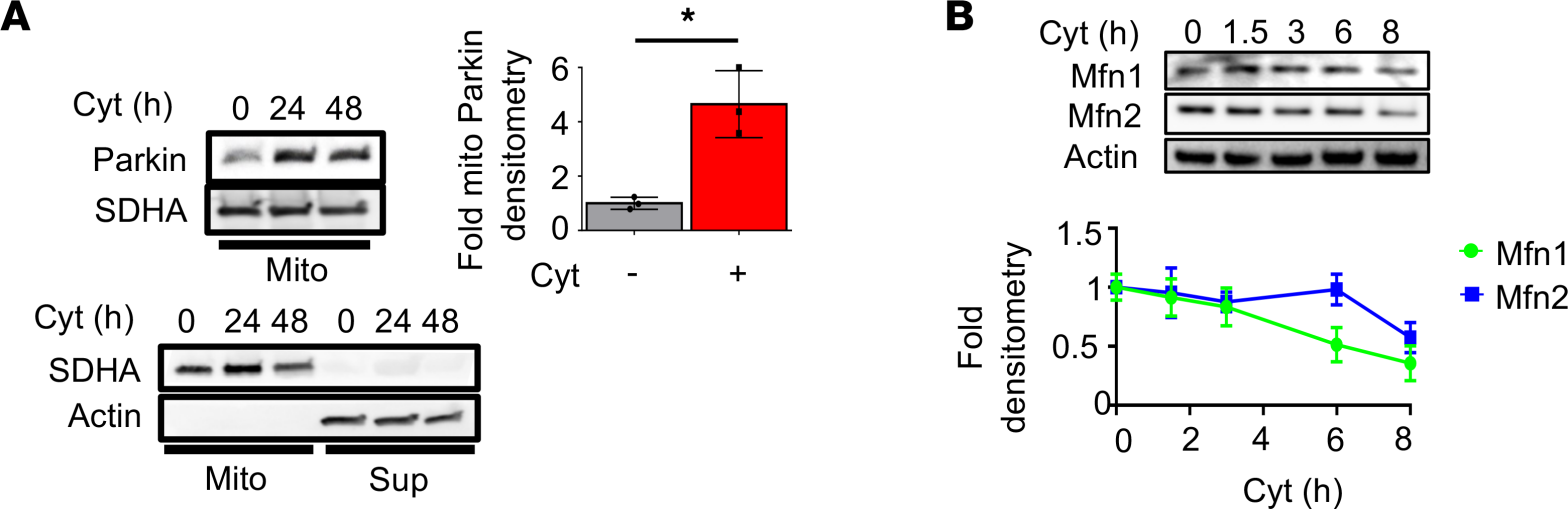
Proinflammatory cytokines induce mitochondrial Parkin translocation and turnover of its outer mitochondrial membrane targets. (**A**) (Top) Mitochondrial Parkin localization by Western blot (WB), with densitometry normalized to SDHA as a loading control in cytokine-treated Min6 β cells following biochemical fractionation of mitochondria. (Bottom) WB following cell fractionation and 8000*g* centrifugation of cytokine- or vehicle-treated Min6 β cells probed for SDHA (mitochondria; mito) or actin (supernatant; sup) to detect purity of mitochondrial fraction. *n* = 3/group. *P < 0.05 by 2-tailed *t* test. (**B**) Mfn1 and Mfn2 expression by WB (with densitometry normalized to actin) in Min6 β cells treated with cytokines for indicated time course. *n* = 3/group (*P* < 0.05 by ANOVA).

The initiation of mitophagy is marked by recruitment of the cytosolic E3 ligase Parkin to depolarized mitochondria, resulting in turnover of outer mitochondrial membrane (OMM) proteins including mitofusins 1 and 2 (Mfn1 and Mfn2, respectively), turnover of Parkin itself, and then clearance of damaged mitochondria by the autophagosome-lysosome pathway (16). In Min6 β cells exposed to inflammatory cytokines, endogenous Parkin translocated to the mitochondria (Figure 2A). Furthermore, we observed a time-dependent decrease of Mfn1 and Mfn2 protein following cytokine exposure (Figure 2B). Classical inducers of mitophagy, including FCCP and valinomycin, induced similar turnover of Mfn1 and Mfn2 protein (Supplemental Figure 2A). Importantly, cytokines induced β cell mitophagy but not bulk macroautophagy; we neither observed differences in the protein levels or cleavage/activation of LC3 (Supplemental Figure 2B), nor in the protein levels of the autophagy substrate p62 following cytokine exposure in mouse islets (data not shown).

**Figure 3:**
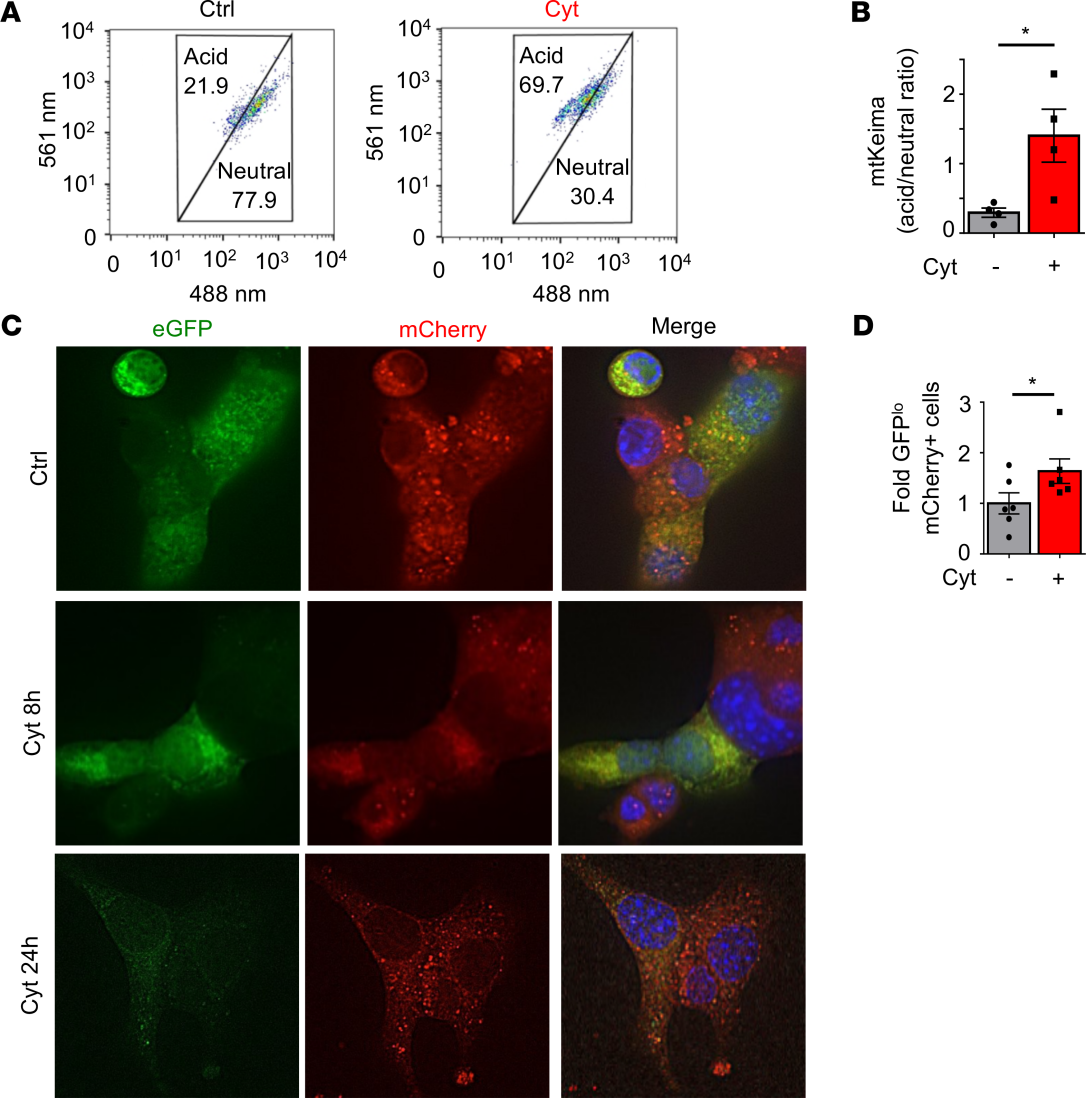
Mitophagy is activated by proinflammatory cytokines. (**A**) Flow cytometry scatter plot from FVB/N mt-Keima islets treated with control or cytokines for 6 hours. Cells gated in upper acid group represent populations with increased relative acidic 561 nm to neutral 480 nm excitation, consistent with activation of mitophagy. Representative of 4 independent experiments. (**B**) Quantification of flow cytometry data by acid/neutral population ratio from FVB/N mt-Keima islets in **A** treated with control or cytokines for 6 hours. *n* = 4/group. *P < 0.05 by 2-tailed *t* test. (**C**) Deconvolution immunofluorescence images at 100× magnification of live Min6 β cells transfected with mitochondria-targeted tandem mCherry-eGFP plasmid following control or cytokine exposure for indicated time course. Nuclei are stained by Hoechst 33342 (blue). Representative of 6 independent experiments. (**D**) Flow cytometric quantification of eGFP and mCherry fluorescence of live Min6 β cells transfected as in **C** following control or cytokine treatment for 24 hours. Data expressed as fold change of eGFP^lo^ and mCherry^+^ cell populations to indicate mitophagy. *n* = 6/group. *P < 0.05 by one-tailed *t* test.

To further assess mitophagy following cytokine exposure, we utilized fluorescently labeled and pH-sensitive mitochondrial biosensors to analyze the translocation of mitochondria to acidic lysosomes via 2 independent and previously validated approaches (18, 19). We first analyzed mitophagy rates in primary islets isolated from mt-Keima mice, which express a mitochondria-targeted reporter that exhibits a shift in excitation/emission spectra based on changes in pH (19). Notably, mt-Keima mice do not exhibit any defects in glucose tolerance when compared with littermate transgene–negative controls (data not shown). Flow cytometry of dissociated islets revealed that cytokine exposure increased the number of cells whose mitochondria were localized to acidic compartments (Figures 3, A and B, and Supplemental Figure 3). As a complementary approach, we expressed a mitochondria-targeted tandem mCherry-eGFP reporter in Min6 β cells (Figure 3C). In cells expressing this reporter, both eGFP and mCherry are detectable in mitochondria at neutral pH, but when mitochondria are in acidic lysosomes, the eGFP fluorescence is quenched (18). Following cytokine exposure, we observed a reduction in eGFP but not mCherry fluorescence, consistent with localization of damaged mitochondria to lysosomes (Figure 3, C and D).

As β cells can recover from inflammatory damage following the withdrawal of cytokines (20), we asked if Δψ_m_ and mitophagy would return to baseline following removal of inflammatory stress. Indeed, cytokine withdrawal rescued Δψ_m_ in WT mouse islets and normalized rates of mitophagy in mt-Keima mouse islets (Supplemental Figure 4, A and B). Taken together, these studies indicate that β cells activate mitophagy in response to inflammatory stress.

### NO and/or ROS are inflammatory mediators of cytokine-induced mitophagy.

**Figure 4:**
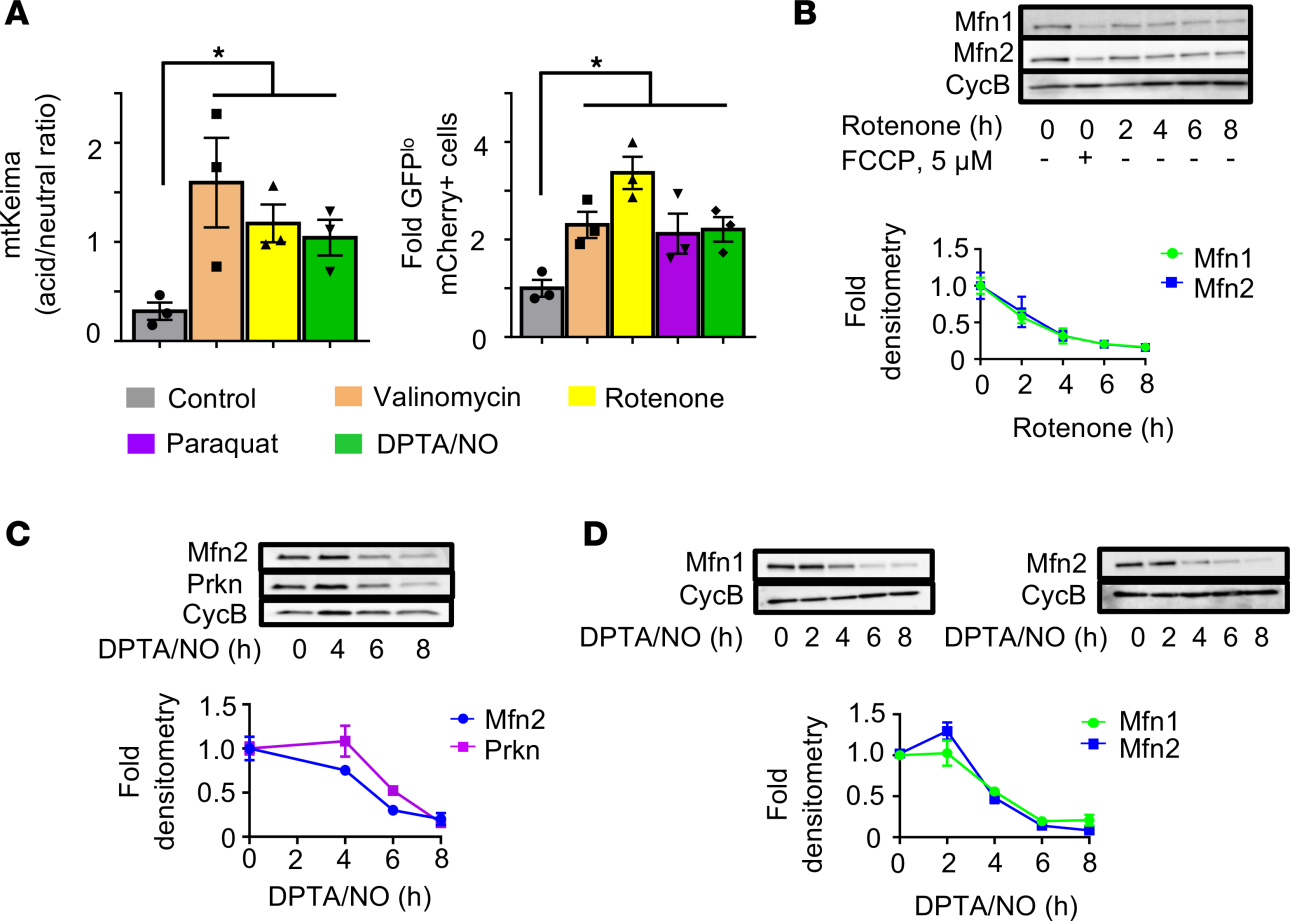
Nitric oxide/reactive oxygen species induce mitophagy in β cells. (**A**) (Left) Assessment of mitophagy by flow cytometric quantification of acid/neutral ratio from FVB/N mt-Keima islets following treatment with valinomycin (250 nM for 3 hours), rotenone (250 nM for 3 hours), and DPTA/NO (600 μM for 1 hour). *n* = 3/group. *P < 0.05 by ANOVA. (Right) Flow cytometric quantification of eGFP and mCherry fluorescence of Min6 β cells transfected with mitochondria-targeted tandem mCherry-eGFP mitophagy reporter following treatment with valinomycin (250 nM for 4 hours), rotenone (500 nM for 4 hours), paraquat (1 mM for 4 hours), and DPTA/NO (600 μM for 6 hours). *n* = 3/group; *P < 0.05 by ANOVA. (**B**) Mfn1 and Mfn2 expression by WB (with densitometry normalized to cyclophilin B) in Min6 β cells treated with 5 μM FCCP for 6 hours or 500 nM rotenone for indicated time course. *n* = 3/group; *P* < 0.05 by ANOVA. (**C**) Mfn2 and Parkin (Prkn) expression by WB (with densitometry normalized to cyclophilin B) in mouse islets treated with DPTA/NO (600 μM) for indicated time course. *n*=4/group; *P* < 0.05 by ANOVA. (**D**) Mfn1 and Mfn2 expression by WB (with densitometry normalized to cyclophilin B) in Min6 β cells treated DPTA/NO (600 μM) for indicated time course. *n* = 3/group; *P* < 0.05 by ANOVA.

Proinflammatory cytokines activate signaling cascades that have both protective and detrimental outcomes. Among the most well-known effectors of inflammatory cytokines are NO and ROS (6). To determine the role of free radicals in the induction of mitophagy, we treated mt-Keima islets and tandem mito-mCherry-GFP–expressing Min6 β cells with pharmacological agents to increase NO or ROS, including DPTA/NO (a NO donor), paraquat (which induces mitochondrial superoxide production), or rotenone (a mitochondrial complex I inhibitor that induces ROS formation). In both islets and Min6 β cells, induction of NO or ROS activated mitophagy (Figure 4A) to a similar extent as valinomycin, a potassium ionophore that induces mitophagy by dissipating Δψ_m_. Furthermore, we observed a concordant decrease in OMM proteins and total Parkin levels upon exposure to rotenone and/or DPTA/NO, again consistent with the induction of β cell mitophagy by ROS or NO (Figure 4, B–D).

**Figure 5:**
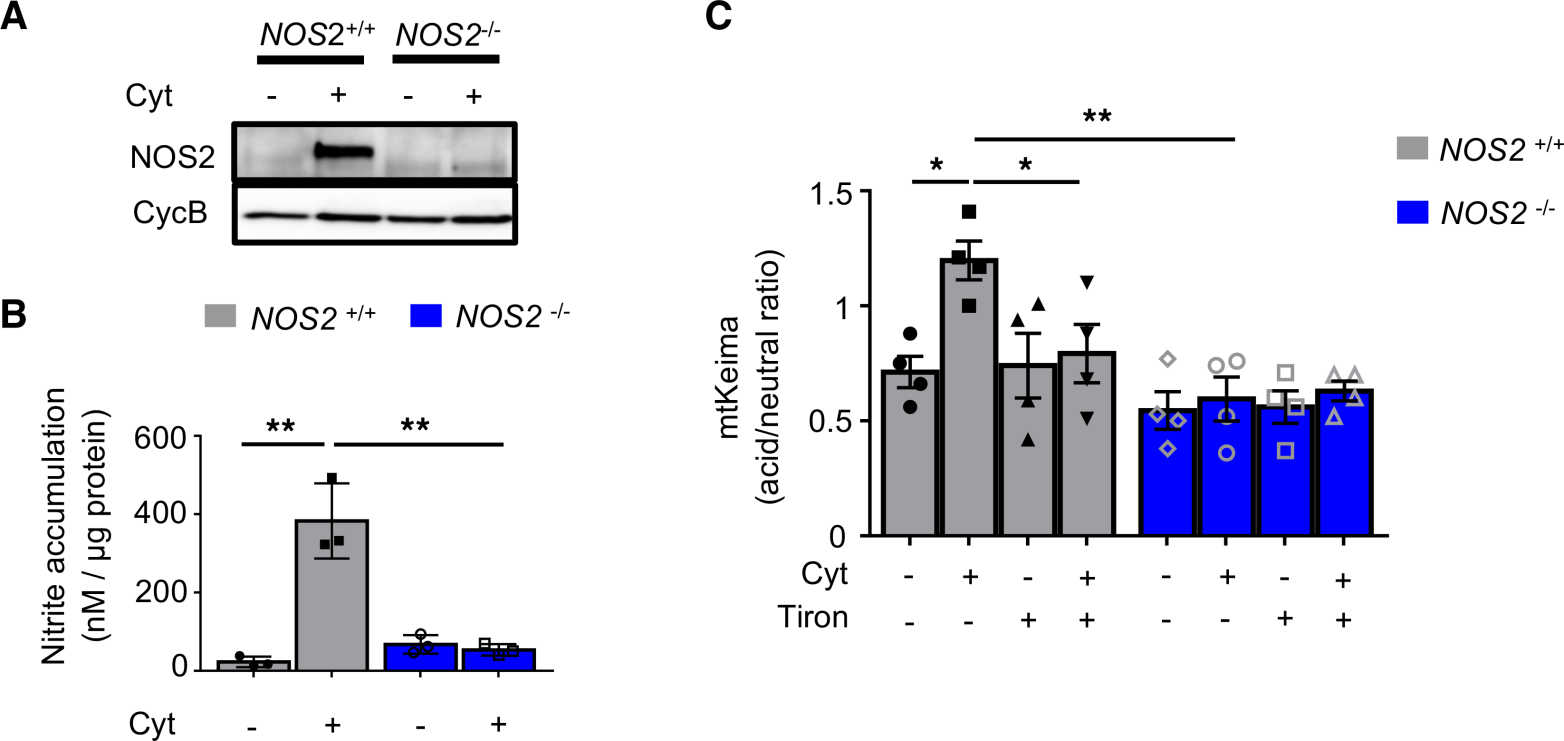
Cytokine-induced free radicals elicit mitophagy in β cells. (**A**) NOS2 expression by WB in WT and *NOS2*^–/–^ islets treated with Ctrl (PBS) or Cyt for 24 hours. Representative of 3 independent mice/group. (**B**) Secreted nitrite concentrations (normalized to islet total protein content) in supernatant collected from cultured WT and NOS2^–/–^ islets treated with PBS or Cyt for 24 hours. *n* = 3/group. **P < 0.01 by ANOVA. (**C**) Assessment of mitophagy by flow cytometric quantification of acid/neutral ratio from WT;mt-Keima (gray) and *NOS2*^–/–^ mt-Keima (blue) islets treated with 500 μM tiron for 24 hours with cytokines for the final 6 hours. *n* = 4/group. *P < 0.05, **P < 0.01 by ANOVA.

To determine if inflammatory cytokines induce mitophagy via free radical generation, we utilized both genetic and pharmacologic approaches. β Cells generate cytokine-induced NO via inducible NO synthase (iNOS, encoded by *NOS2*; refs. 21, 22); therefore, we intercrossed *NOS2*^–/–^ mice and mt-Keima mice for mitophagy analysis. As expected, loss of iNOS decreased NO release following cytokine exposure in islets (Figure 5, A and B). In addition, cytokine-mediated induction of mitophagy was abrogated in the islets of *NOS2*^–/–^ mt-Keima mice (Figure 5C). Similarly, treatment with the mitochondria-permeable superoxide scavenger tiron (23) prevented activation of cytokine-induced mitophagy in WT mt-Keima islets (Figure 5C). Of note, tiron may have effects to scavenge NO in addition to superoxide (24, 25). Thus, our results indicate that cytokine-induced free radicals elicit mitophagy in β cells.

### Impaired β cell mitophagy exacerbates hyperglycemia and mitochondrial fragmentation in vivo following inflammatory stimuli.

**Figure 6:**
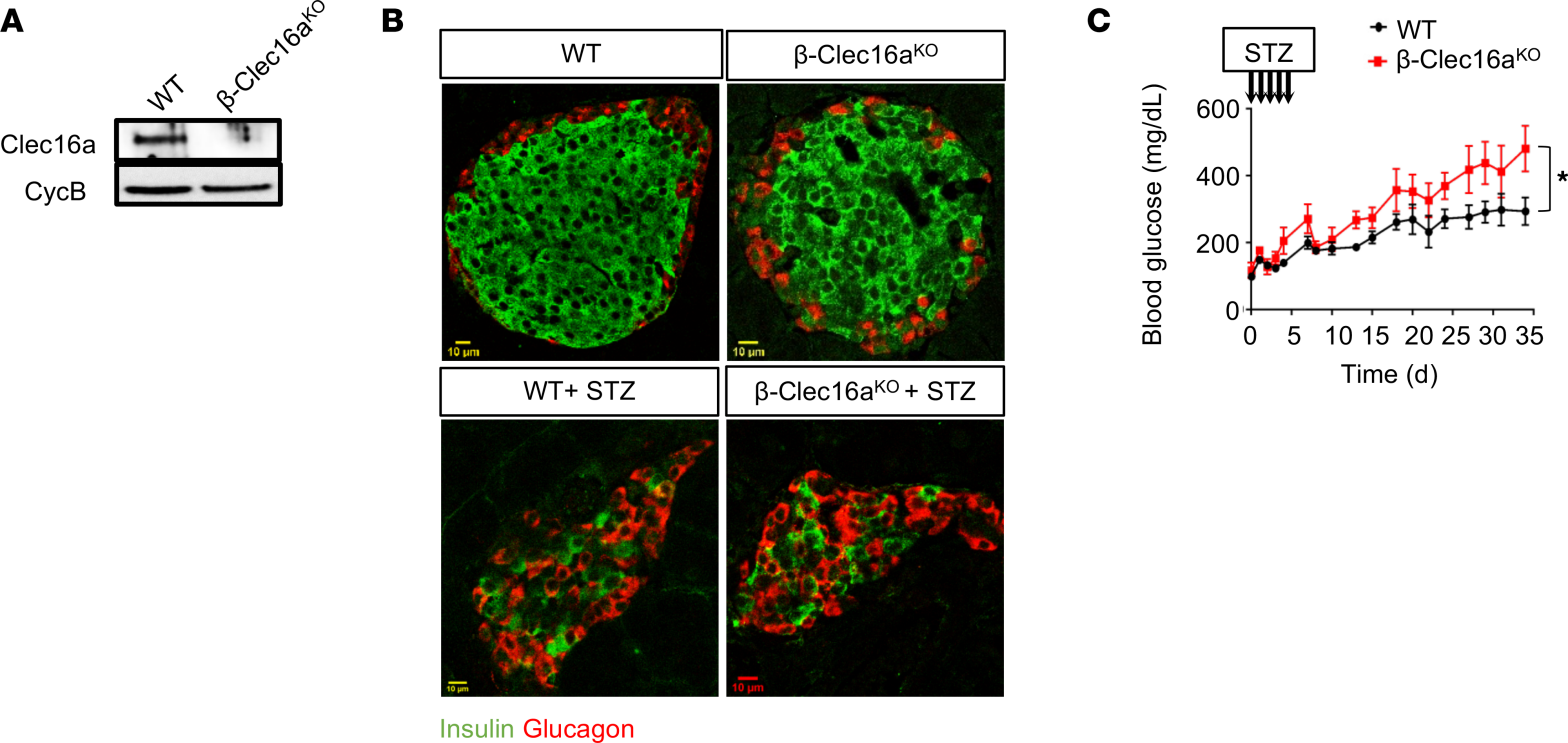
Mitophagy deficiency aggravates STZ-induced dysglycemia. (**A**) Clec16a expression by WB in 10-week-old WT and β-Clec16a^KO^ mouse islets. cyclophilin B serves as a loading control. Representative of 4 independent mice/group. (**B**) Confocal immunofluorescence images at 60× magnification of insulin (green) and glucagon (red) from pancreatic sections of WT and β-Clec16a^KO^ mice, in the presence or absence of STZ treatment (50 mg/kg/d i.p. for 5 days). Representative of 5–8 independent mice/group. (**C**) Randomly fed blood glucose concentrations from WT (black) and β-Clec16a^KO^ mice (red) following STZ treatment. *n* = 5–8/group. *P < 0.05 by ANOVA.

Our observation that mitophagy is activated following inflammatory β cell damage led us to hypothesize that mitophagy is a protective response that preserves β cell function in the setting of inflammatory stress. To interrogate the role of mitophagy in vivo, we deleted the key mitophagy regulator Clec16a specifically in β cells (*Clec16a*^loxP/loxP^;*Ins1*-Cre, hereafter referred to as β-Clec16a^KO^; Figure 6A). *Clec16a* encodes an E3 ubiquitin ligase vital for the clearance of dysfunctional β cell mitochondria via mitophagy (11, 12). We previously demonstrated that Clec16a tunes mitophagic flux through formation of a tripartite Clec16a-Rnf41(Nrdp1)-Usp8 complex that both restrains activation of Parkin-mediated mitophagy during physiologic states and promotes autophagosome- or mitophagosome-lysosome fusion to ensure the completion of mitophagy following mitochondrial damage (11–13, 26). As expected, unchallenged β-Clec16a^KO^ mice did not exhibit defects in randomly fed blood glucose values and only mild glucose intolerance after an i.p. glucose challenge (data not shown), consistent with our previous reports (11, 13).

To determine the importance of mitophagy in β cell inflammatory responses, we treated β-Clec16a^KO^ mice and littermate controls with multiple low doses (50 mg/kg/d × 5 days) of the β cell toxin and inflammatory stressor streptozotocin (STZ) (27–29). Both WT and β-Clec16a^KO^ mice showed a reduction in β cells in response to STZ (Figure 6B). However, the effect of STZ on blood glucose levels was significantly exacerbated in β-Clec16a^KO^ mice (Figure 6C), supporting a role for mitophagy in preserving β cell function following inflammatory stress.

**Figure 7:**
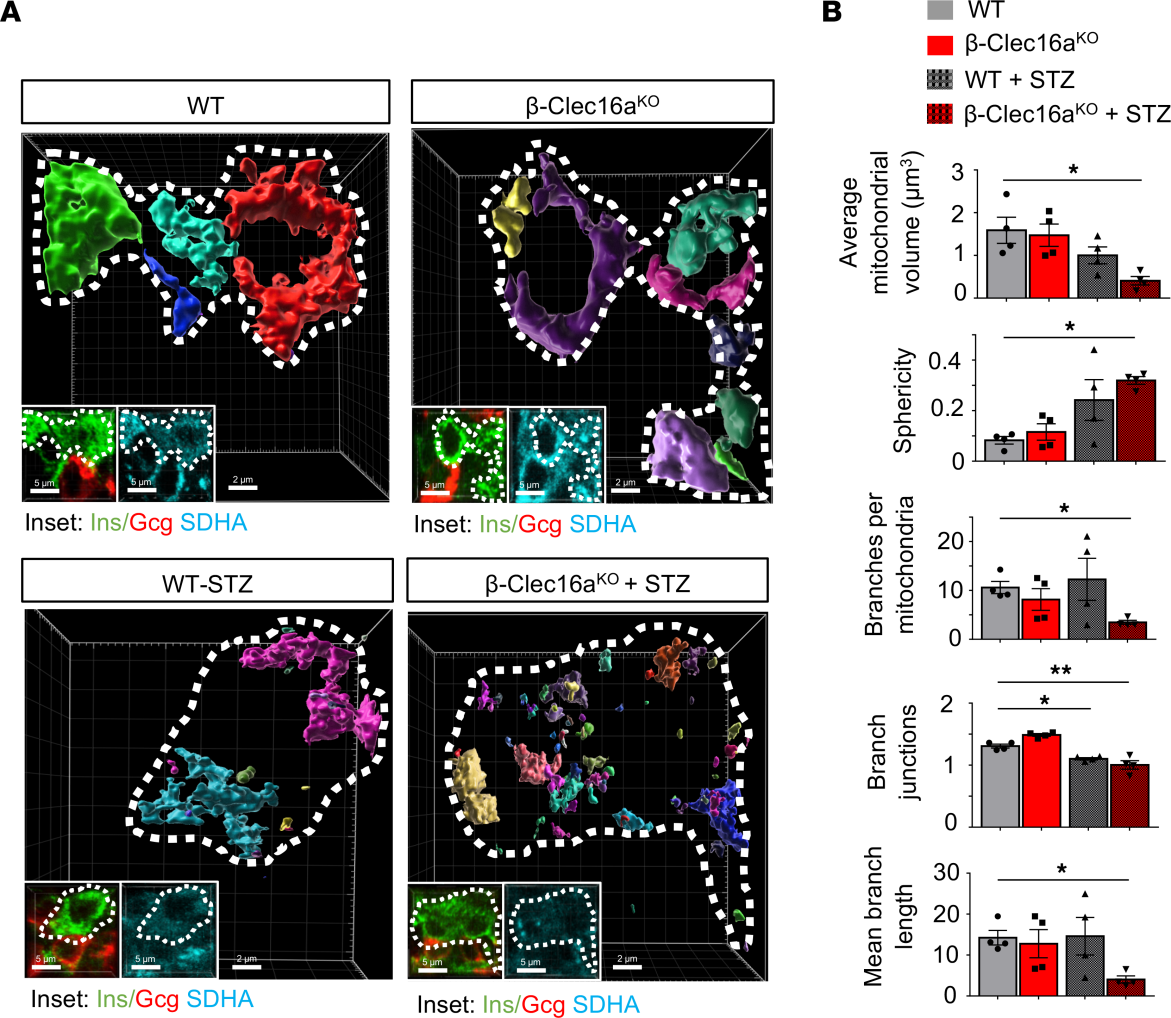
Mitophagy deficiency reveals STZ-induced mitochondrial network defects in β cells in vivo. (**A**) Imaris generated 3D reconstruction of deconvolution immunofluorescence *Z*-stack images at 100× magnification stained for SDHA (see inset image; blue) from pancreatic sections of WT and β-Clec16a^KO^ mice, in the presence or absence of STZ treatment. β Cells and α cells were identified by costaining (inset: insulin, Ins [green]; glucagon, Gcg [red]). β Cells are encircled by a dotted line (white). Each unique color represents a separate β cell mitochondrial network cluster. Representative image of 4 independent mice/group. (**B**) β Cell mitochondrial morphology and network analysis of deconvolution immunofluorescence *Z*-stack images from studies depicted in **A**, stained for SDHA (and insulin) from pancreatic sections of WT and β-Clec16a^KO^ mice, in the presence or absence of STZ treatment by MitoAnalyzer. *n* = 4/group (185–220 β cells/animal were quantified). *P < 0.05, **P < 0.01 by ANOVA.

Damaged mitochondria are segregated from the mitochondrial network by fission, leading to a fragmented appearance of the network prior to the elimination of dysfunctional mitochondria by mitophagy (30). We hypothesized that inflammatory stress due to STZ would induce mitochondrial fragmentation, which would be exacerbated in mitophagy-deficient β cells. We thus examined mitochondrial morphology and networks in islets of β-Clec16a^KO^ mice and littermate controls in the presence or absence of STZ. Utilizing confocal imaging of the mitochondrial marker succinate dehydrogenase A (SDHA), we generated 3-dimensional (3D) reconstructions of islet mitochondria and quantified mitochondrial morphology and network appearance. In the absence of STZ, mitochondria from control and β-Clec16a^KO^ β cells revealed relatively similar 3D mitochondrial appearance between genotypes (Figure 7A). STZ treatment, in contrast, increased the frequency of smaller vesiculated mitochondrial networks in WT β cells, consistent with fragmentation; however, mitochondrial fragmentation was dramatically exacerbated by the deletion of Clec16a (Figure 7A). 3D quantification confirmed that STZ treatment altered mitochondrial network complexity in control β cells by decreasing mitochondrial branch junctions (Figure 7B). Mitochondrial morphology and network/branch complexity markedly worsened in STZ-treated β-Clec16a^KO^ β cells, consistent with an accumulation of fragmented mitochondria due to deficient mitophagy. Together, these data support an important role for mitophagy in protecting β cell mitochondrial networks from inflammatory stimuli in vivo.

### Clec16a regulates cytokine-induced mitophagy in rodent and human islets.

**Figure 8:**
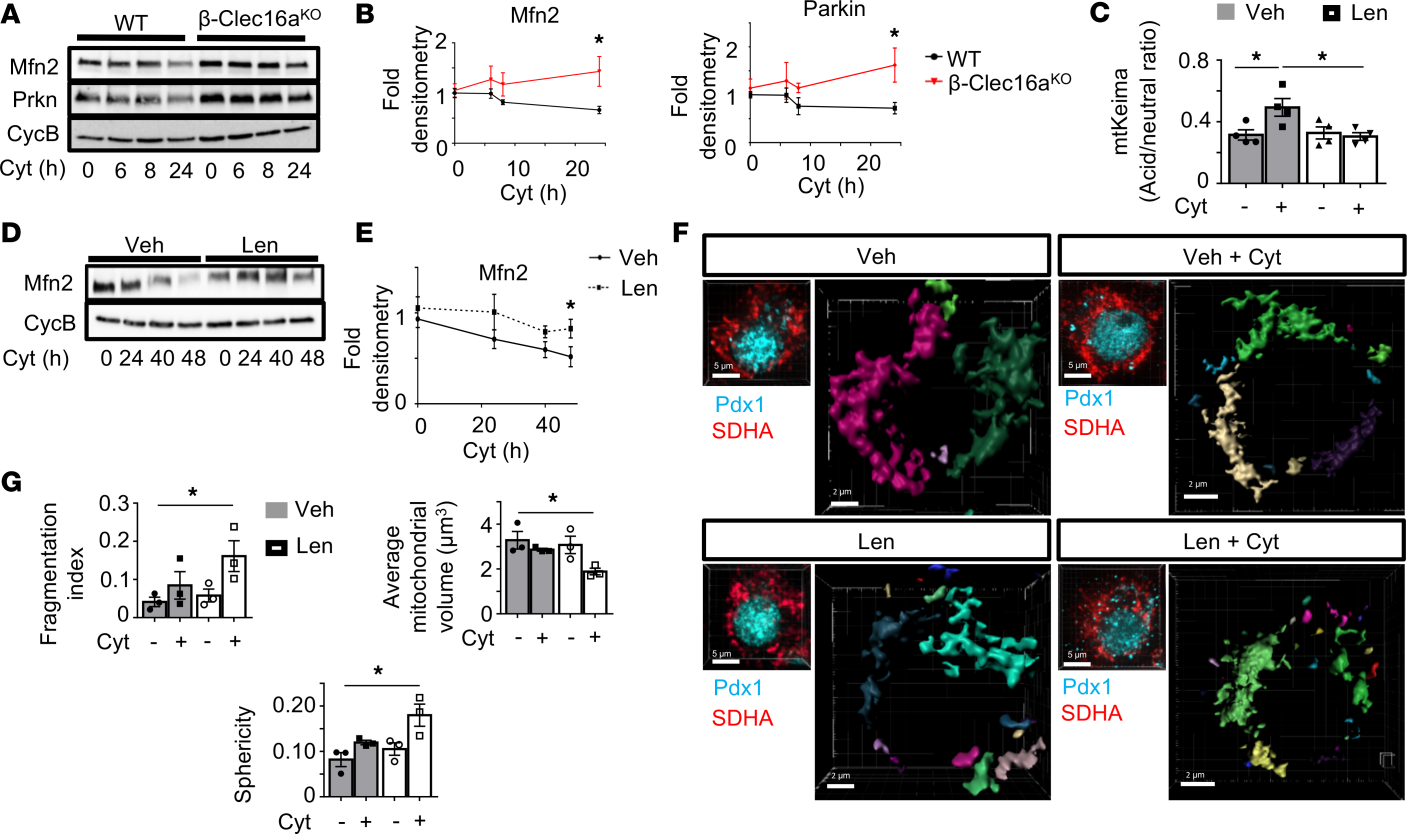
Clec16a regulates cytokine-induced mitophagy in human and rodent islets. (**A**) Mfn2 and Prkn expression by WB in WT and β-Clec16a^KO^ islets treated with cytokines for indicated time course. *n* = 4/group. (**B**) Mfn2 and Prkn densitometry (normalized to cyclophilin B) from studies in **A**. *n* = 4/group; * *P* < 0.05 versus WT 24 hours by 2-tailed *t* test. (**C**) Assessment of mitophagy by flow cytometric quantification of acid/neutral ratio from B6N mt-Keima islets treated with vehicle (Veh; DMSO) or 10 μM lenalidomide (Len) for 24 hours with cytokines for the final 6 hours. *n* = 4/group. *P < 0.05 by ANOVA. (**D**) Mfn2 expression by WB in human islets treated with Veh or 10 μM Len for 72 hours in the presence of cytokines for the final 48 hours per indicated time course. *n* = 4/group. (**E**) Mfn2 densitometry (normalized to cyclophilin B) from studies in **D**. *n* = 4/group; * *P* < 0.05 versus Veh 48 hours by 2-tailed *t* test. (**F**) Imaris generated 3D renderings of deconvolution immunofluorescence *Z*-stack images at 100× magnification stained for SDHA (see offset images; red) in human β cells. β Cells were identified by Pdx1 costaining (see offset images; blue). Each unique color represents a separate β cell mitochondrial network cluster. *n* = 3/group. (**G**) Human β cell mitochondrial morphology and network analysis of confocal immunofluorescence *Z*-stack images stained for SDHA from studies depicted in **F** by MitoMap and MitoAnalyzer. *n* = 3/group (75–110 β cells from each donor per condition were quantified). *P < 0.05 by ANOVA.

The accumulation of fragmented mitochondria in β cells from STZ-treated Clec16a-deficient mice led us to speculate that Clec16a regulates mitochondrial turnover following exposure to proinflammatory cytokines. Indeed, β-Clec16a^KO^ islets had impaired clearance of Mfn2 and Parkin following cytokine exposure, consistent with Clec16a control of cytokine-induced mitophagy (Figure 8, A and B). Similarly, treatment of islets from mt-Keima mice with lenalidomide, a pharmacologic inhibitor of the CLEC16A-mediated mitophagy pathway (11, 31), impaired cytokine-induced mitophagy (Figure 8C).

To interrogate mitophagy as a response to inflammatory damage in human islets, we assessed several complementary mitochondrial endpoints. Cytokine exposure led to reduced Mfn2 protein levels, as well as increased localization of mitochondria (marked by SDHA) within lysosomes (marked by LAMP1), consistent with the activation of mitophagy (Figure 8, D and E, and Supplemental Figure 5, A and B). Cytokine-induced mitophagy was also blocked by lenalidomide treatment (Figure 8, D and E, and Supplemental Figure 5, A and B). Furthermore, the combination of cytokines and lenalidomide increased mitochondrial fragmentation (Figure 8, F and G, and Supplemental Figure 6). Therefore, in both human and rodent β cells, CLEC16A-mediated mitophagy protects mitochondrial network integrity following inflammatory stress.

### Mitophagy deficiency exacerbates cytokine-induced β cell death.

**Figure 9:**
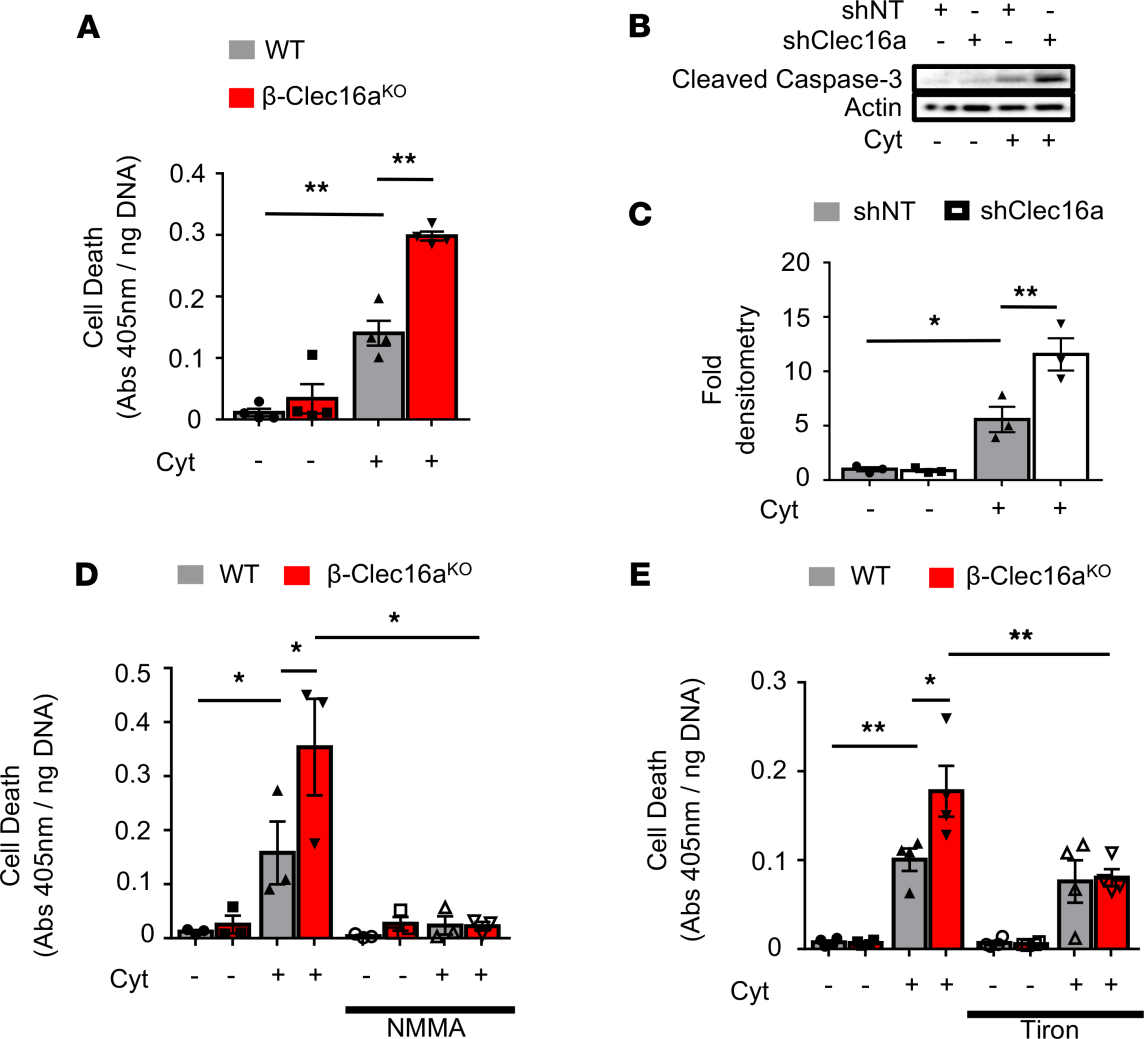
Mitophagy is a protective response to cytokine-induced nitrosative/oxidative stress. (**A**) Quantification of cell death by cytoplasmic histone–complexed DNA fragment ELISA (normalized to total DNA content) in WT and β-Clec16a^KO^ islets treated with/without cytokines for 24 hours. *n* = 4/group. **P < 0.01 by ANOVA. (**B**) Cleaved caspase 3 expression by WB in nontargeting (NT) or Clec16a-specific shRNA expressing Min6 β cells treated with cytokines for 6 hours. Representative of 3 independent experiments. (**C**) Cleaved caspase 3 densitometry (normalized to actin) from studies depicted in **B**. *n* = 3/group. *P < 0.05, **P < 0.01 versus by ANOVA. (**D**) Quantification of cell death by cytoplasmic histone–complexed DNA fragment ELISA (normalized to total DNA content) in WT and β-Clec16a^KO^ islets treated with/without NMMA (500 μM) for 48 hours and treated with/without cytokines for the final 24 hours. *n* = 3/group; *P < 0.05 by ANOVA. (**E**) Cell death–detection ELISA (normalized to total DNA content) in WT and β-Clec16a^KO^ islets treated with/without 500 μM tiron for 48 hours and treated with/without cytokines for the final 24 hours. *n* = 4/group. *P < 0.05, **P <0.01 by ANOVA.

While we demonstrated that mitophagy preserved mitochondrial networks following inflammatory injury, it was unclear if mitophagy also preserved β cell viability after inflammatory damage. To address this question, we assessed apoptosis in Clec16a-deficient β cells (and controls) following cytokine exposure. Indeed, cytokine exposure induced increased cell death in β-Clec16a^KO^ islets compared with controls (Figure 9A). Cytokine exposure similarly increased cell death in Min6 β cells following shRNA-mediated Clec16a knockdown (Figures 9, B and C, and Supplemental Figure 7A). Of note, cytokine exposure did not affect Clec16a expression (Supplemental Figure 7A).

Next, we utilized pharmacologic agents targeting NO/ROS to determine if free radicals contribute to the enhanced cytokine-induced apoptosis we observed in mitophagy-deficient islets. Indeed, treatment with the NOS inhibitor L-NMMA abrogated cytokine-induced apoptosis in both control and β-Clec16a^KO^ islets (Figure 9D). We also observed that enhanced cell death in β-Clec16a^KO^ islets was ameliorated by tiron (Figure 9E). Thus, mitophagy promotes β cell survival in response to nitrosative/oxidative stress.

We next asked whether mitophagy-deficient β cells are sensitized to cytokine-mediated apoptosis due to increased formation of free radicals. Interestingly, loss of Clec16a led to reduced, not increased, cytokine-induced nitrite levels compared with controls (Supplemental Figure 7B). Furthermore, Clec16a loss of function did not affect baseline or cytokine-induced ROS levels or expression of transcriptional targets associated with inflammatory NF-κB signaling (Supplemental Figure 7, C and D). Moreover, Clec16a-deficiency did not affect basal or cytokine-induced cellular import of iron, a key catalyst of ROS production (Supplemental Figure 7E). Similarly, inhibition of CLEC16A with lenalidomide did not affect cytokine-induced expression of NF-κB targets in human islets (Supplemental Figure 7, F and G). Thus, we speculate that mitophagy mediates β cell mitochondrial responses to nitrosative/oxidative stress but does not directly affect the signaling pathways leading to free radical formation.

### CLEC16A overexpression protects human β cells from cytokine toxicity.

**Figure 10:**
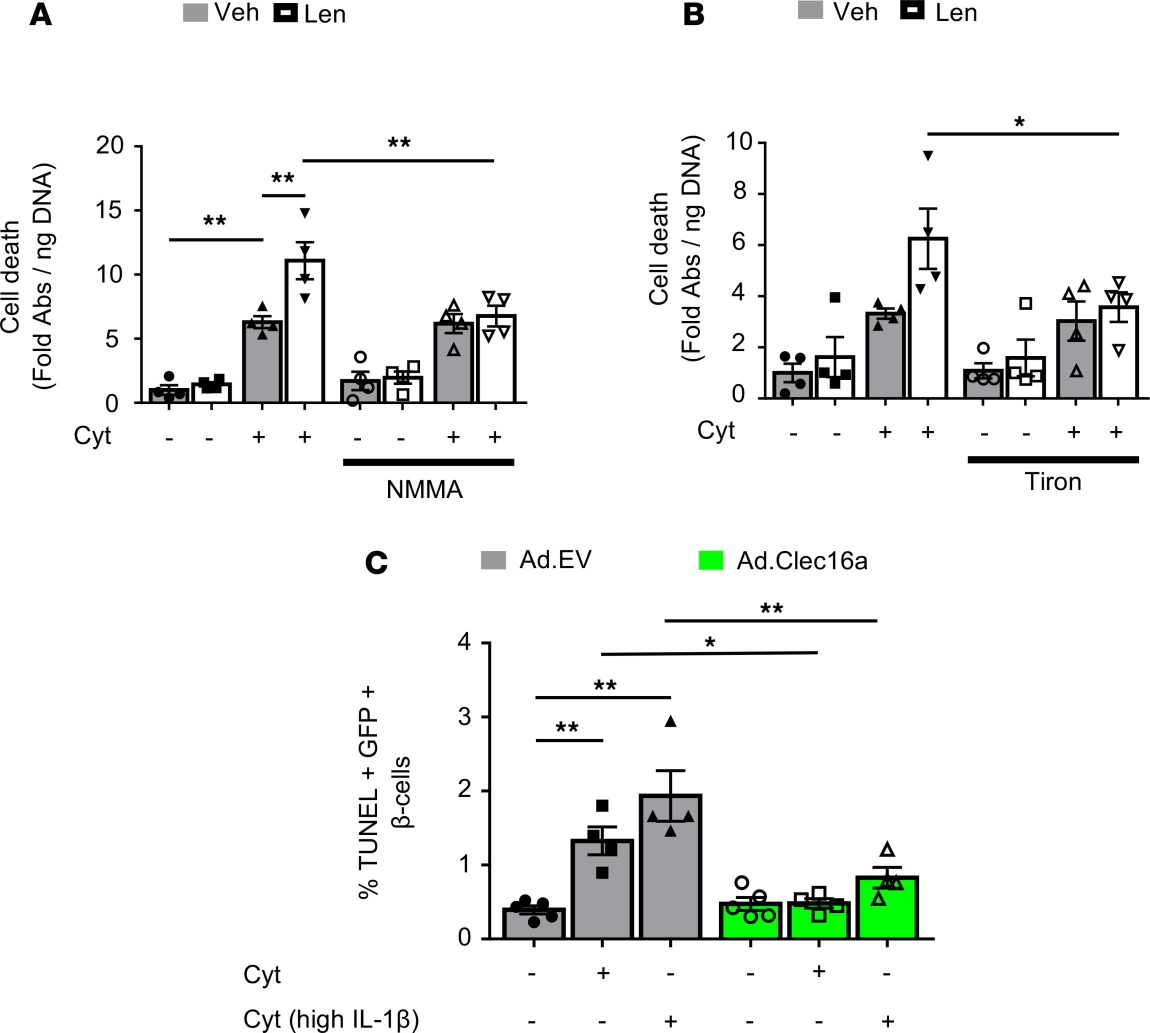
CLEC16A protects human β cells from cytokine-induced apoptosis. (**A**) Cell death–detection ELISA in human islets treated with/without 10 μM lenalidomide and/or 500 μM NMMA for 48 hours and then treated with/without cytokines for the final 24 hours. *n* = 4/group; **P < 0.01 by ANOVA. (**B**) Cell death–detection ELISA in human islets treated with/without 10 μM lenalidomide and/or 500 μM tiron for 48 hours and then treated with/without cytokines for the final 24 hours. *n* = 4/group; *P < 0.05 by 2-tailed *t* test. (**C**) Quantification of %TUNEL^+^GFP^+^ β cells by immunofluorescence staining on human islets transduced with empty vector control (Ad.EV; black) or Clec16a-overexpressing (Ad.Clec16a; green) adenoviral particles, following treatment with/without cytokines for 24 hours. β Cells were identified by insulin immunostaining, and transduced cells were identified by GFP immunostaining. Cyt group treated with 75 U/mL IL-1β, 750 U/mL TNF-α, and 750 U/mL IFN-γ. The Cyt^hi^ IL-1β group was treated with 3000 U/mL IL-1β, 750 U/mL TNF-α, and 750 U/mL IFN-γ. *n* = 4-5/group. *P < 0.05, **P < 0.01 by ANOVA.

Our observations of enhanced cytokine-mediated apoptosis in mitophagy-deficient mouse islets led us to hypothesize that CLEC16A protects against β cell death in human islets. Indeed, we observed that CLEC16A inhibition by lenalidomide enhanced cytokine-induced cell death in human islets (Figure 10A). In addition, NO/ROS blockade specifically ameliorated lenalidomide-related cell death but did not affect vehicle-treated human islets (Figure 10, A and B). To test whether CLEC16A overexpression could protect against human β cell apoptosis, we transduced intact human islets with adenoviruses overexpressing CLEC16A or an empty vector control, as well as IRES-eGFP, selectively in β cells (Supplemental Figure 8, A and B). Remarkably, CLEC16A overexpression significantly prevented cytokine-mediated apoptosis in human β cells, even in the setting of higher IL-1β concentrations (Figure 10C and Supplemental Figure 8C). We did not observe any differences in β cell viability in vehicle-treated human islets transduced with CLEC16A compared with empty vector, suggesting that the protective benefits of CLEC16A overexpression were observed following inflammatory injury (Figure 10C). We also observed a similar protective effect of Clec16a overexpression in Min6 β cells following cytokine exposure (Supplemental Figure 9, A and B). These results indicate that the mitophagy regulator CLEC16A protects human β cells against inflammatory injury.

## Discussion

Here, we identify mitophagy as a vital protective response to inflammatory assault of human β cells. We demonstrate that inflammatory cytokines activate the cardinal steps of mitophagy, including the dissipation of Δψ_m_, mitochondrial translocation of Parkin, turnover of OMM proteins, mitochondrial segregation, and mitochondrial localization to lysosomes for their elimination. We show that cytokine-mediated mitophagy is activated in response to free radicals. Moreover, we identified a protective role for the T1D gene *CLEC16A* in preventing β cell death following inflammatory stress.

Our results indicate that, by controlling mitophagy, CLEC16A may be a key determinant of β cell susceptibility to inflammation-induced apoptosis. Recent reports position CLEC16A as the principal mediator of T1D risk at the chromosome 16.13 locus (13, 32–34). While *CLEC16A* polymorphisms in T1D are associated with reduced human islet CLEC16A expression, as well as impaired β cell function and glycemic control, prior studies have not clarified the importance of CLEC16A in β cells following diabetogenic stressors. Importantly, we observe that CLEC16A deficiency sensitizes β cells to inflammatory stress, which may provide insight into how *CLEC16A* polymorphisms increase susceptibility to the development of T1D. Future studies in autoimmune diabetes models and samples from donors with T1D will be necessary to further clarify β cell–specific roles for CLEC16A in T1D. However, our findings lead us to speculate that CLEC16A deficiency may be a novel mediator of β cell fragility (35, 36), which has been recently appreciated as a component of T1D pathogenesis.

*CLEC16A* is ubiquitously expressed and also has key functions in immune cells (37–42). Recent work demonstrating mitochondrial metabolic defects linked to inflammatory responses in immune cells of T1D donors may suggest an unexplored role for CLEC16A-mediated mitophagy in T1D (7, 43). Likewise, defective mitophagy has been associated with elevated secretion of cytokines by macrophages (44, 45). Together with our studies in β cells, CLEC16A-mediated mitophagy may play pleotropic roles in multiple cell types in T1D and therefore may represent a potent therapeutic target.

Our studies are the first to our knowledge to show a specific link between mitophagy and protection against inflammatory β cell death. Autophagy comprises several pathways that maintain cellular homeostasis, including selective and nonselective/bulk macroautophagy, as well as microautophagy and chaperone-mediated autophagy (46). Previous studies on macroautophagy demonstrate a cytoprotective role in β cells (47, 48), but these studies primarily relied upon β cell–specific deletion of proteins that broadly impair both selective and nonselective autophagy. Therefore, the importance of nonselective versus selective autophagy in β cells has remained unresolved. Nonselective macroautophagy is classically induced by nutrient deprivation (49), whereas diabetes is a disease of glucose/nutrient excess, suggesting an underappreciated role for other forms of autophagy in β cell function and survival. Importantly, our data demonstrate a specific induction of mitophagy (and not bulk macroautophagy) by inflammatory damage, potentially placing selective autophagy in a key position in the development of diabetes.

Our studies demonstrate a protective role of CLEC16A-mediated mitophagy in human β cells exposed to inflammatory stimuli and support the feasibility of therapeutically targeting this pathway. Several mitophagy-activating compounds have been recently found to improve respiratory function in metabolic tissues, including β cells (50–53). Indeed, the mitophagy activator urolithin A has shown great promise in support of whole-body metabolic function in clinical trials (54). Therefore, pharmacologic enhancement of mitophagy could represent a novel future approach to prospectively compensate for inflammatory stress and prevent β cell death in diabetes. Future studies will be required to fully characterize the efficacy of targeting mitophagy in the treatment of all forms of diabetes.

## Methods

### Animals.

Mouse models included *Clec16a*^loxP^ (13), *Ins1*-Cre (55), mt-Keima (ref. 19 and a gift from Toren Finkel, University of Pittsburgh, Pittsburgh, Pennsylvania, USA), and *NOS2*^–/–^ mice (56). mt-Keima mice were separately maintained on the FVB/N and C57BL/6N (B6N) backgrounds, while all other models were maintained on the B6N background. *Clec16a*^loxP^ were mated to *Ins1*-Cre mice, and B6N mt-Keima were mated to *NOS2*^–/–^ mice to generate experimental groups. For studies using the conditional Clec16a allele, *Ins1*-Cre–alone mice and *Clec16a*^loxP/loxP^ mice were phenotypically indistinguishable from each other and, thus, combined as WT controls. For STZ studies, 5-week-old male mice were injected with 50 mg/kg STZ (Cayman Chemical) for 5 consecutive days (days 1–5). Randomly fed blood glucose concentrations were measured 3–4 times weekly for up to 35 days after STZ. Animals were housed on a standard 12-hour light/12-hour dark cycle with ad libitum access to food and water.

### Islet isolation and cell culture.

Islets were isolated from male and female mice between the ages of 8 and 15 weeks, and they were cultured as previously described (11, 13). Min6 β cells (passages 28–40) were maintained as previously described (11, 13). Human islets were acquired from the Integrated Islet Distribution Program (IIDP) and cultured as previously described (11). Donor information is provided in Supplemental Table 1.

### Cell treatments, transfections, and viral transductions.

Min6 β cells and mouse islets were exposed to a cytokine combination consisting of murine IL-1β, TNF-α, and IFN-γ (Peprotech) for 6–24 hours. Human islets were treated with a cytokine mixture containing human IL-1β, TNF-α, and IFN-γ (Peprotech) for 24–48 hours. Additional cell treatments included dimethylsulfoxide as a vehicle control (DMSO; Thermo Fisher Scientific), lenalidomide (MilliporeSigma), tolbutamide (MilliporeSigma), FCCP (MilliporeSigma), valinomycin (MilliporeSigma), rotenone (MilliporeSigma), paraquat (MilliporeSigma), DPTA/NO (Cayman Chemical), L-NMMA (Cayman Chemical), and tiron (MilliporeSigma). Min6 β cells were transfected using an Amaxa Nucleofector (Lonza) as previously described (13). The tandem mitochondrial-targeted mCherry-eGFP mitophagy reporter plasmid (pCLBW-cox8-EGFP-mCherry) was a gift from David Chan (Caltech, Pasadena, California, USA; Addgene, 78520). pLKO.1-shNT, pLKO.1-shClec16a, pFLAG-CMV5a-empty vector, and pFLAG-CMV5a-Clec16a constructs were previously described (11, 13) and used for knockdown or overexpression studies. Adenoviral vectors and particles were generated (VectorBuilder) expressing Clec16a-3xFLAG (Ad.Clec16a) or an empty vector control (Ad.EV), as well as an IRES-GFP, which were driven by the 2.9 kb Pdx1 proximal promoter sequence (57) for β cell–selective expression. Intact human islets (100 islet equivalents [IEQs]) were transduced with 1 × 10^9^ PFU of Ad.Clec16a or Ad.EV for 24 hours, prior to cytokine exposure 48 hours after infection for cell survival studies. β Cell transduction was confirmed by insulin and GFP costaining. Human islets were transduced overnight with adenoviral particles expressing the PercevalHR biosensor (described below) 48 hours prior to cytokine exposure (58).

### ROS, NO, and iron measurements.

ROS levels were measured by DCF fluorescence (Abcam) as described (59). Nitrite levels were detected in supernatant culture media and measured using Griess reagents as described (60). NO/ROS measurements were normalized to total protein content (MicroBCA; Thermo Fisher Scientific). Cellular labile iron pools were detected as described (61), imaged by confocal microscopy after loading with 1 μM calcein-AM (Invitrogen) and again following chelation with 300 μM deferasirox (MilliporeSigma).

### Western blot, reverse transcription, quantitative PCR, cell fractionation, and immunostaining.

All assays were performed as previously described (11, 13). Commercially available antibodies used for Western blotting and immunostaining are listed in Supplemental Table 2. Rabbit Clec16a-specific polyclonal antisera (Cocalico) were generated following inoculation with a recombinant Clec16a protein fragment (AA347-472). Primer sequences used for quantitative reverse transcription PCR (qRT-PCR) are provided in Supplemental Table 3. See complete unedited blots in the supplemental material.

### Flow cytometry.

Following exposures and treatments, live mt-Keima mouse islets were dissociated into single cells by the use of 0.25% trypsin-EDTA (Thermo Fisher Scientific) for 1 minute at 37°C. Single cells were stained with DAPI (Thermo Fisher Scientific) and resuspended in phenol red–free RPMI1640 medium supplemented with 100 units/mL antimycotic-antibiotic (Thermo Fisher Scientific), 50 units/mL penicillin-streptomycin (Thermo Fisher Scientific), 1 mM sodium pyruvate (Thermo Fisher Scientific), and 10 mM HEPES (Thermo Fisher Scientific). Samples were analyzed on an LSR Fortessa flow cytometer (BD Biosciences). Single cells were gated using forward scatter and side scatter (FSC and SSC, respectively) plots, and DAPI staining was used to exclude dead cells. Dual-excitation ratiometric mt-Keima measurements were made using 488 nm (neutral) and 561 nm (acidic) excitation lasers with 610 nm emission filter (62, 63). Ratios of acidic to neutral cell populations were then calculated using FlowJo (Tree Star Inc.). A total of 5,000 islet cells was quantified from each independent islet preparation. Gating strategies are found in Supplemental Figure 3.

Live Min6 β cells transfected with tandem mitochondrial-targeted mCherry-eGFP mitophagy reporter (18) were detached from culture plates with the use of 0.25% trypsin-EDTA for 1 minute, resuspended into single cells by gentle pipetting in phenol red–free DMEM media (Thermo Fisher Scientific) supplemented with 50 units/mL penicillin-streptomycin (Thermo Fisher Scientific), 1 mM sodium pyruvate (Thermo Fisher Scientific), and 142 μM β-mercaptoethanol and then stained with DAPI. Samples were then analyzed on a ZE5 flow cytometer (Bio-Rad). Single cells were gated using FSC and SSC plots, and DAPI staining was used to exclude dead cells. Mitochondrial fluorescence in cell populations was assessed using 488 nm (GFP) and 561 nm (mCherry) excitation lasers with 525 nm and 610 nm emission filter sets, respectively. Live cell populations were then gated for mCherry^+^ cells that contained low GFP fluorescence (FlowJo) to quantify mitophagy. A total of 10,000 cells was quantified from each independent sample.

### Mitochondrial bioenergetic measurements.

Oxygen consumption was measured using oxygen microsensing glass electrodes (UniSense). The membrane potential (V_m_) of human islets was measured with patch electrodes having < 10 Mohm resistance connected to micromanipulators and a HEKA USB EPC10 patch-clamp amplifier (Heka Instruments) in the perforated patch-clamp configuration. Islets were perifused with a standard external solution containing 135 mM NaCl, 4.8 mM KCl, 3 mM CaCl_2_, 1.2 mM MgCl_2_, 20 mM HEPES, 10 mM glucose (pH 7.35). When higher KCl solutions were used, NaCl was reduced equimolar for KCl. β Cells were identified by the presence of characteristic slow oscillations in 10 mM glucose (64). The ATP/ADP ratio was monitored using the PercevalHR biosensor (65). Islets were perifused with 16.7 mM glucose to stimulate ATP production in a recording chamber on an Olympus IX-73 inverted epifluorescence microscope (Olympus). PercevalHR was excited at 488 nm using a TILL Polychrome V monochromator (FEI), and a QuantEM:512SC cooled CCD camera (PhotoMetrics) was used to collect emission at 527 nm (58). Data were acquired and analyzed using Metafluor (Invitrogen) and plotted using Igor Pro (WaveMetrics Inc.).

Live-cell imaging in other studies was performed using a laser-scanning Nikon A1 confocal microscope equipped with an environmental chamber (Tokai Hit) maintained at 37°C and 5% CO_2_. *Z*-stack images were acquired using a Plan Fluor 40× oil objective (Nikon Instruments) and NIS Elements Software (Nikon Instruments). Δψ_m_ was assessed in live human islets using the V_m_-sensitive pentamethinium fluorescent probe, TBMS-306 (66), a gift from Vladimir Kral and Tomas Ruml (University of Chemistry and Technology, Prague, Prague, Czechia). Islets were stained with TBMS-306 (50 nM) for 30 minutes at 37°C and costained with Fluozin-3 (500 nM) to identify β cells, as described (17). *Z*-stack images were acquired using 488 nm (Fluozin-3) and 640 nm (TBMS-306) excitation lasers with 510 nm and 670 nm emission filter sets, respectively.

### Cell death assessments.

Cytoplasmic histone–complexed DNA fragments were determined in islets using the Cell Death ELISAplus (Roche), per the manufacturer’s protocol. Cell pellets were analyzed to detect apoptosis. ELISA absorbance was measured at 450 nm and normalized to total DNA content (SYBRGreen; Invitrogen) using an absorbance/fluorescence microplate reader (BioTek). TUNEL was performed on transduced human islets (Apoptag In situ Apoptosis Detection Kit; Millipore) as per the manufacturer’s protocol. Human islets were prepared for staining as previously described (11). TUNEL-stained samples were counter stained with insulin and GFP antibodies. Images were captured with an IX81 microscope (Olympus) using an ORCA Flash4 CMOS digital camera (Hamamatsu). Apoptotic transduced β cells (TUNEL^+^, insulin^+^, GFP^+^) were counted as a fraction of total transduced β cells (insulin^+^, GFP^+^). DAPI served as a nuclear DNA control.

### Mitochondrial morphology and subcellular localization.

Mitochondrial morphology and localization studies were performed on immunostained human islets or paraffin-embedded mouse pancreas tissue sections, prepared as previously described (11, 13). *Z*-stack images were captured with an IX81 microscope (Olympus) using an ORCA Flash4 CMOS digital camera (Hamamatsu) and subjected to deconvolution (CellSens; Olympus). Colocalization analyses were performed using the Coloc2 plugin on ImageJ (NIH). Mitochondrial morphology was visualized using 3D-renderings generated with Imaris imaging software (Bitplane). Quantitative 3D assessments of mitochondrial morphology and network were performed using the MitoMap and MitoAnalyzer plugins on ImageJ (67, 68).

### Statistics.

In all figures, data are presented as means ± SEM. Statistical comparisons were performed using unpaired 1- or 2-tailed Student’s *t* tests or 1-way ANOVA, followed by Tukey’s post hoc test for multiple comparisons, as appropriate (Prism GraphPad). *P* < 0.05 was considered significant.

### Study approval.

Animal studies were approved by the University of Michigan IACUC. Studies in human islets from deidentified deceased donors were approved by the University of Michigan IRB.

## Author contributions

VS conceived, designed, and performed experiments; interpreted results; and drafted and reviewed the manuscript. GLP, VSP, BT, LC, MAG, JZ, TS, JR, ECR, and BC designed and performed experiments and interpreted results. JAC, TMP, and LSS designed studies, interpreted results, and reviewed the manuscript. SAS conceived and designed the studies, interpreted results, and edited and reviewed the manuscript.

## Supplementary Material

Supplemental data

Supplemental Table 1

Supplemental Table 2

Supplemental Table 3

## Version 1 11/24/2020

In-Press Preview

## Version 2 12/17/2020

Electronic publication
