# Supplementary material for: Mitophagy protects β cells from inflammatory damage in diabetes
Source: JCI Insight. 2020 Dec 17;5(24):e141138. doi: 10.1172/jci.insight.141138 (PMC7819751; doi:10.1172/jci.insight.141138)
Supplement: Supplemental data [file jciinsight-5-141138-s090.pdf]

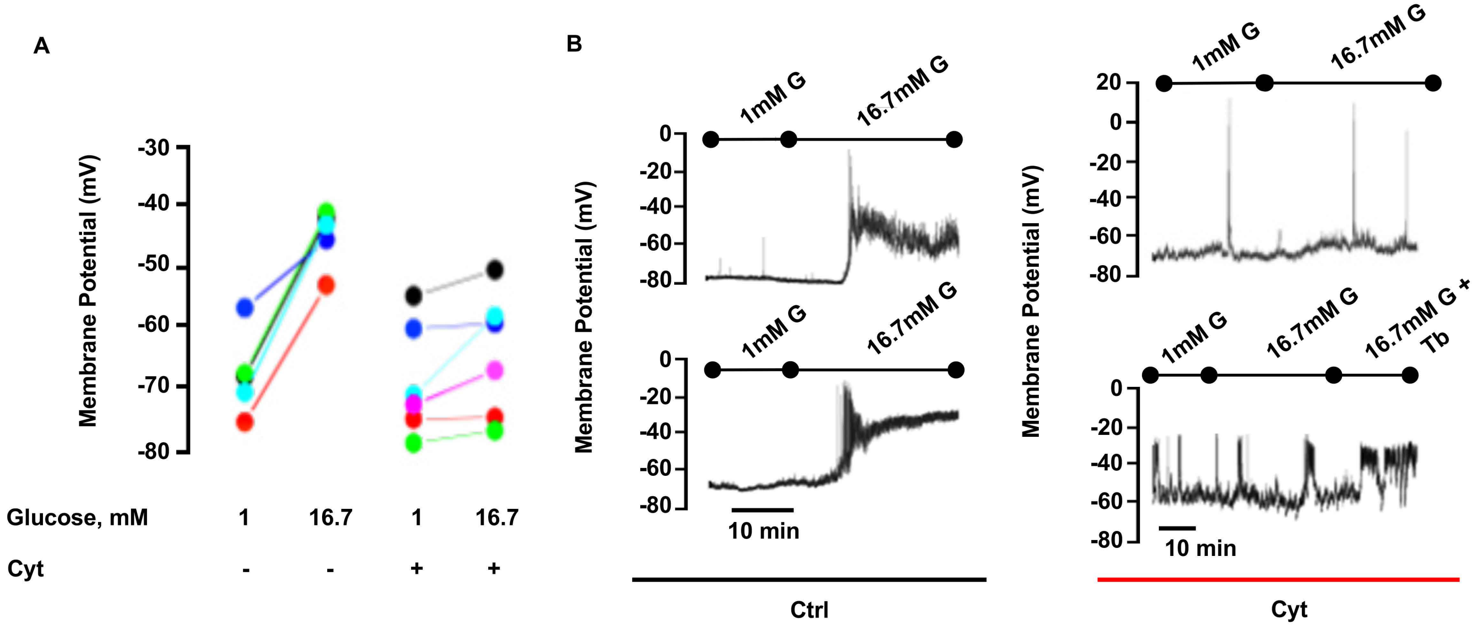

Figure S1. Pro-inflammatory cytokines impair metabolic function in human islets.
(A) Membrane potential assessments by patch clamp in Ctrl and Cyt-treated human islets. Colors represent measurements from independent human islet donors. (B) Representative electrical recordings from Ctrl (left) and Cyt-treated (right) human islets stimulated with glucose and 100μM tolbutamide (Tb) as indicated. n=3-6 independent human islet donors/group for all measurements.

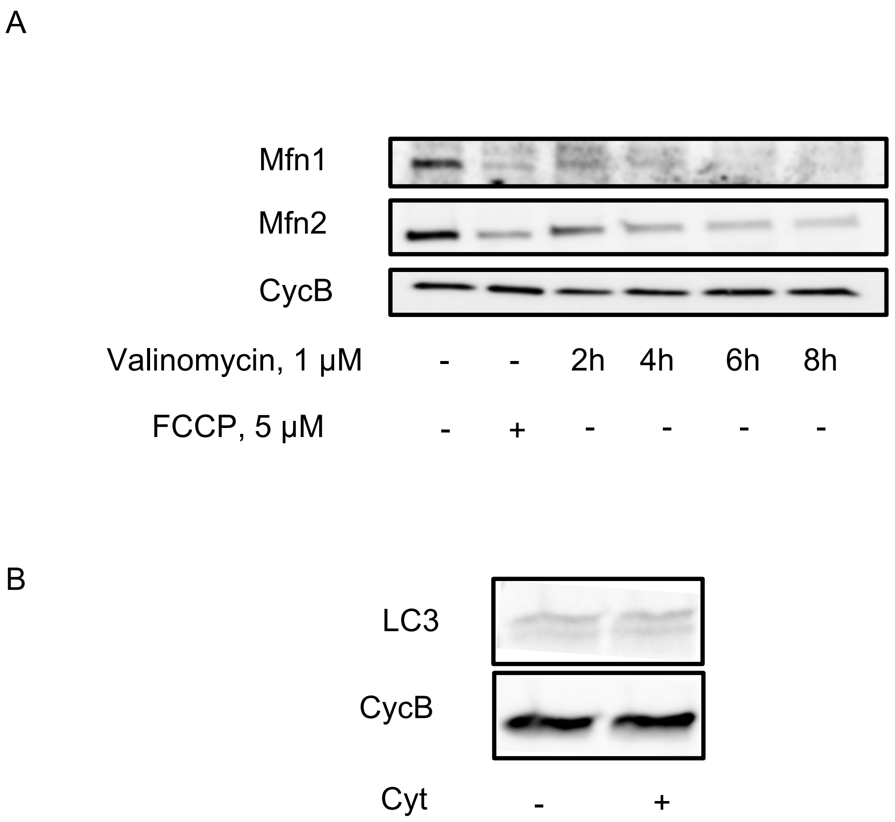

Figure S2. Evaluation of mitophagy and bulk macroautophagy following mitochondrial damage.
(A) Mfn1 and Mfn2 expression by WB in Min6 β-cells treated with 5μM FCCP for 6hrs or 1μM valinomycin for indicated time course. n=3/group. (B) LC3 expression by WB in primary mouse islets treated with cytokines for 24hrs. n=3/group.

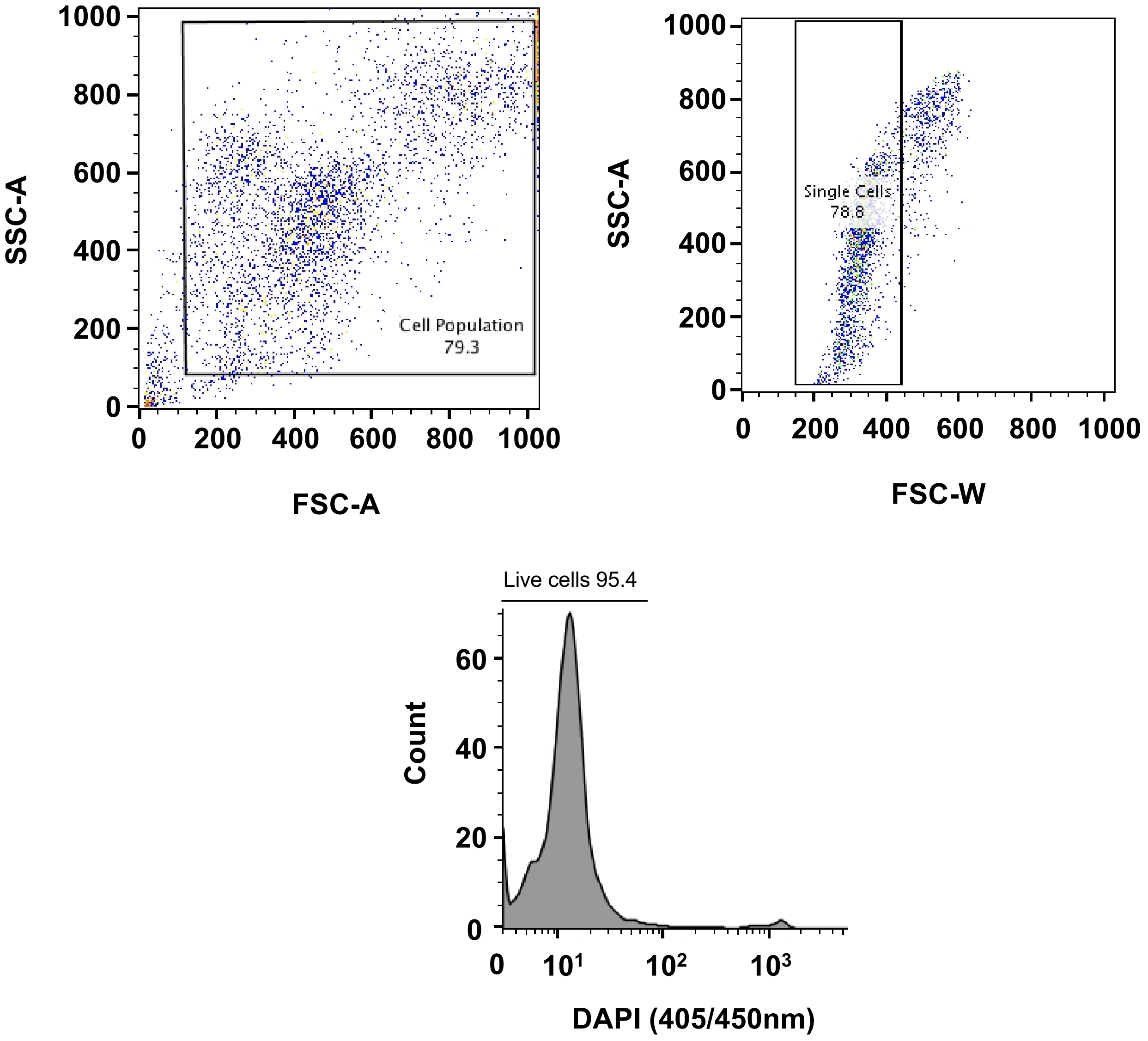

Figure S3. Gating strategies for islet mtKeima flow cytometry studies.
Representative plots generated from dissociated mtKeima mouse islets mitophagy reporter analyzed by flow cytometry. Cells were first gated using FSC-A and SSC-A plots to exclude cell debris. Single cells were then gated using FSC-W and SSC-A plots to exclude cell clumps. DAPI staining was then used to analyze live cell populations.

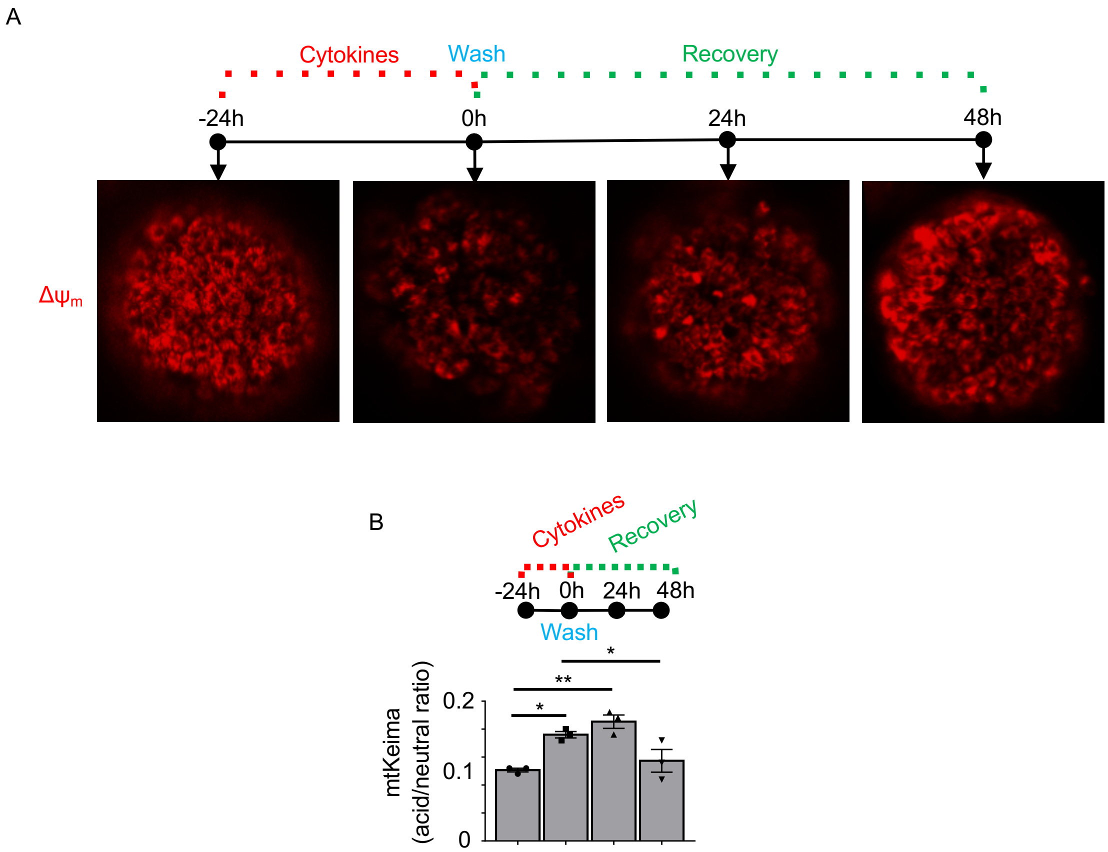

Figure S4. Mitochondrial membrane potential and mitophagy recover after the withdrawal of pro-inflammatory cytokines.
(A) Laser scanning confocal microscopy images at 60X magnification of live B6N mouse islets stained with TBMS-306 ($\Delta\psi_m$) following cytokine treatment for 24hrs and recovery after cytokine removal for 24 and 48hrs. n=3/group. (B) Flow cytometric quantification of acid/neutral population ratio from B6N mtKeima islets following cytokine treatment for 24hrs and recovery after cytokine removal for 24 and 48hrs. n=3/group. *$p<0.05$ ** $p<0.01$ by ANOVA.

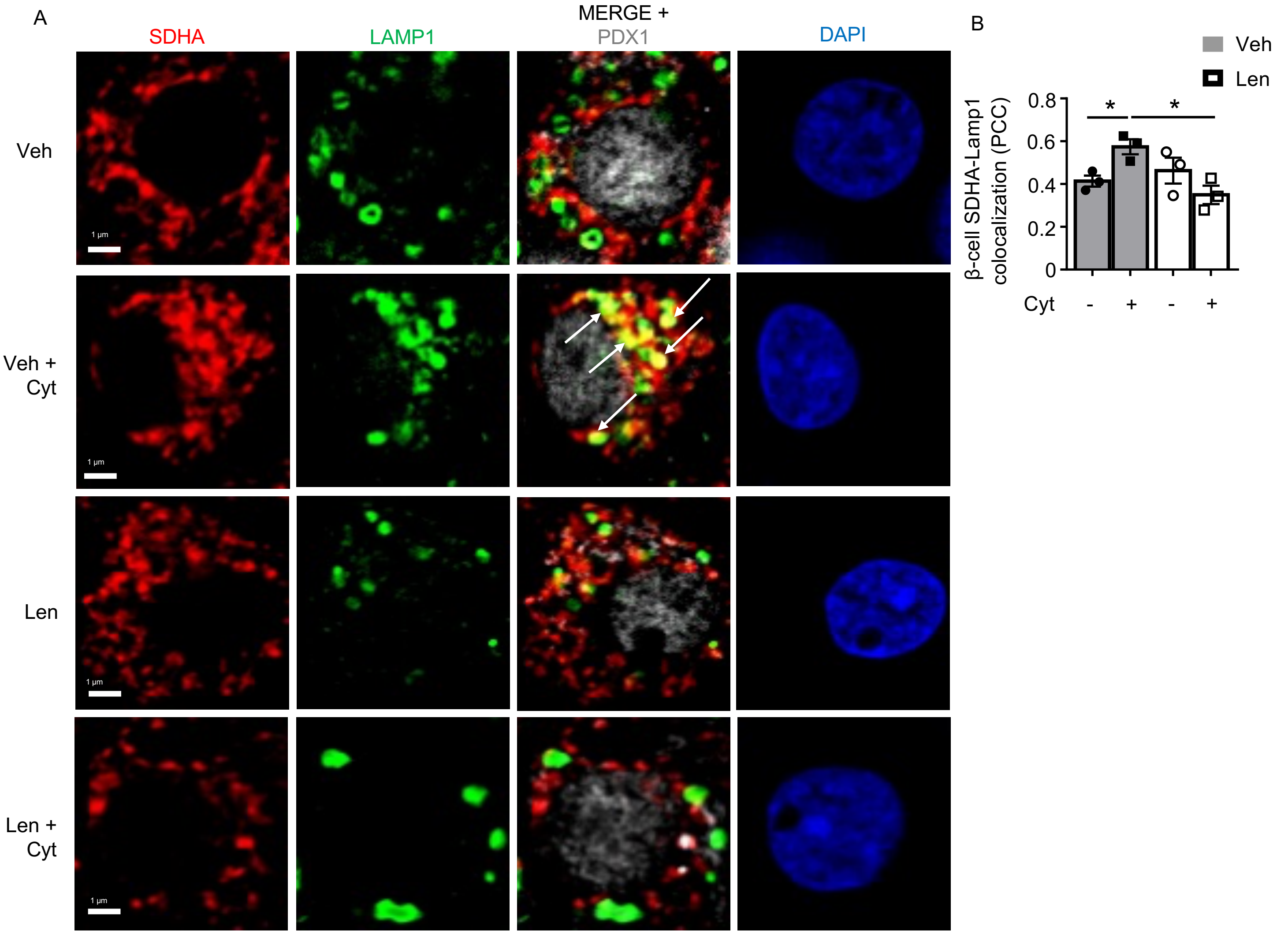

Figure S5. Pharmacologic CLEC16A inhibition impairs cytokine-induced mitophagy in human β-cells.
(A) Deconvolution immunofluorescence image at 100X magnification of human islets treated with Veh or 10μM Len for 72hrs in the presence/absence of cytokines for the final 24hrs stained for SDHA (red), LAMP1 (green), Pdx1 (gray), and DAPI (blue). Representative of 3 independent experiments. (B) Quantification of $Lamp1^{+}SDHA^{+}$ colocalization in human β-cells from studies depicted in Figure S5A by Pearson's correlation coefficient (PCC). n=3/group. * $p<0.05$ by two-tailed t-test. (60-130 β-cells from each donor per condition were analyzed).

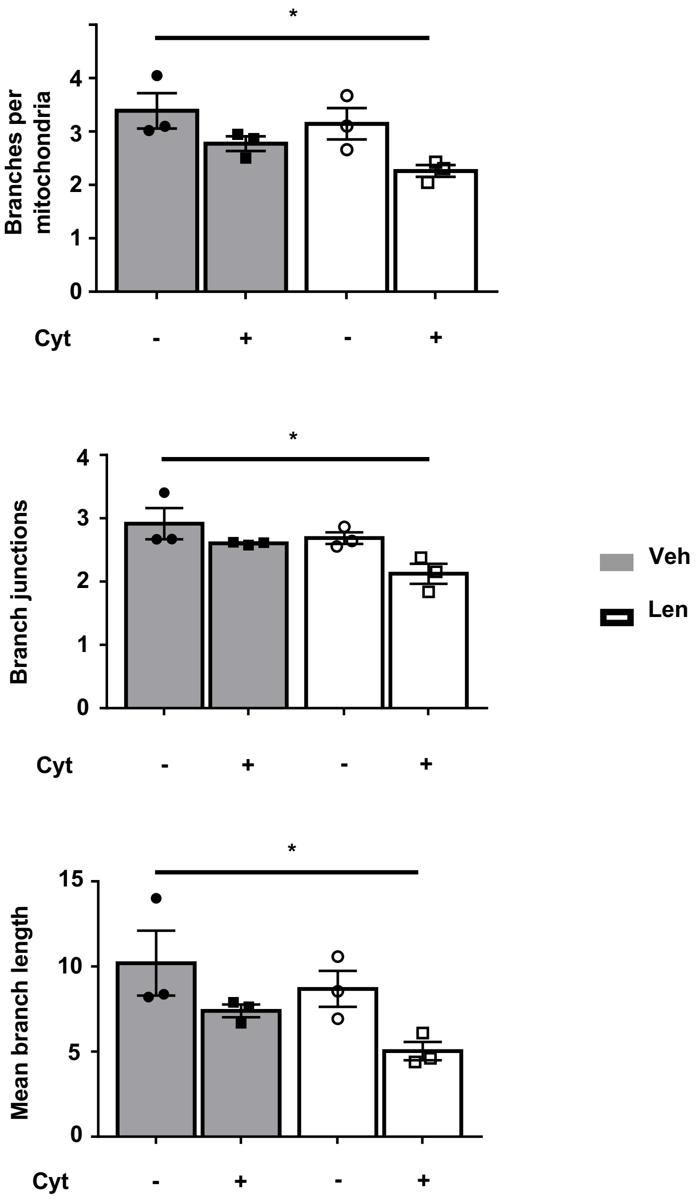

Figure S6. Mitochondrial fragmentation in mitophagy-deficient human β-cells following pro-inflammatory cytokine exposure.

Human β-cell mitochondrial network analysis of confocal immunofluorescence Z-stack images stained for SDHA from studies depicted in Figure 8F by MitoAnalyzer. n=3/group (75-110 β-cells from each donor per condition were quantified). * $p<0.05$ by ANOVA.

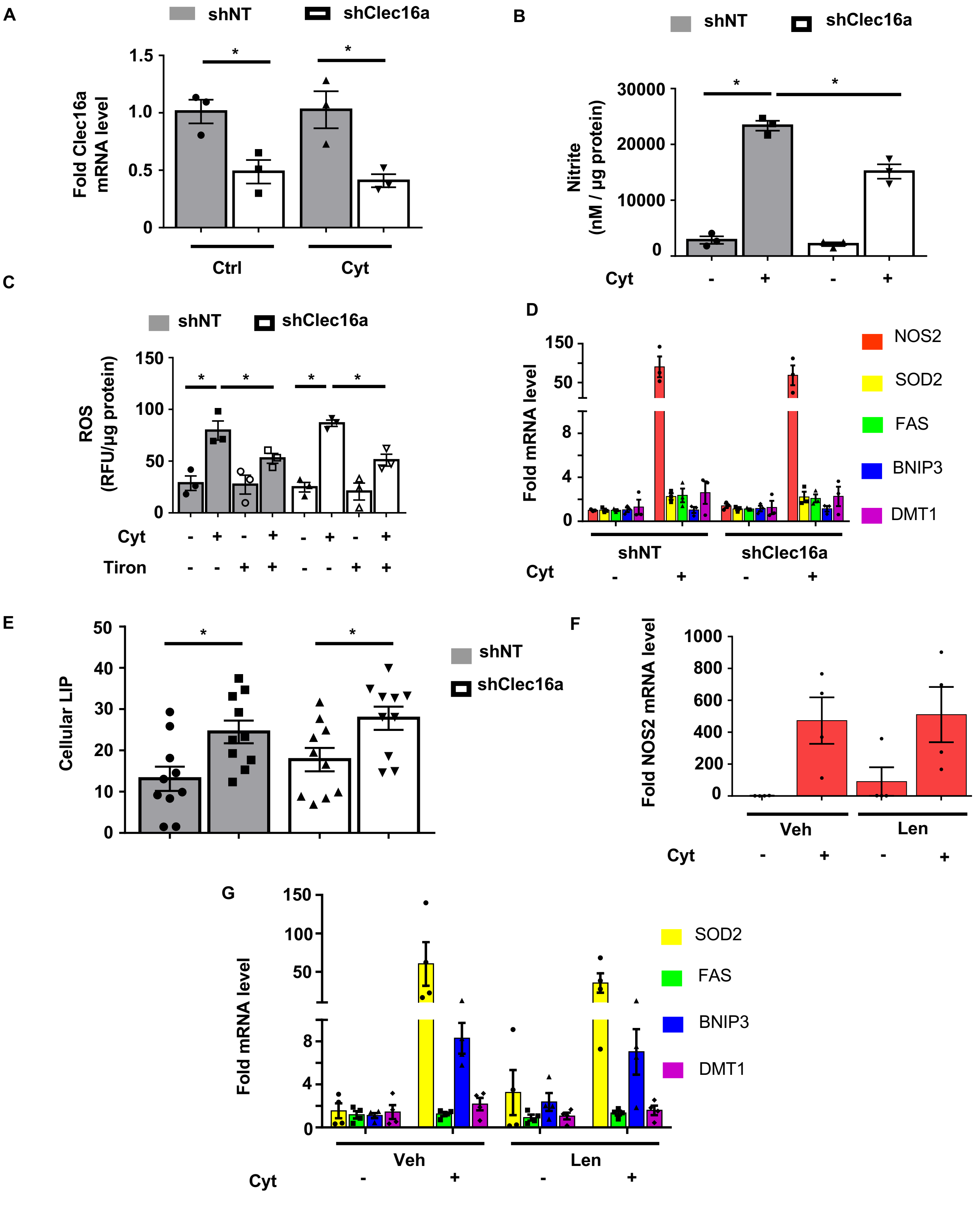

Figure S7. Mitophagy deficiency does not induce differences in inflammatory mediators following cytokine exposure.
(A) Clec16a mRNA expression (normalized to Hprt) by qRT-PCR from RNA isolated from shNT or shClec16a-expressing Min6 β-cells treated in the presence/absence of cytokines for 24hrs. n=3/group. * $p<0.05$ by ANOVA. (B) Nitrite release (normalized to total protein content) in cell culture supernatants of NT or Clec16a-specific shRNA expressing Min6 β-cells treated with/without cytokines for 24hrs. n=3/group; * $p<0.05$ by ANOVA. (C) Total cellular reactive oxygen species (normalized to total protein levels) in NT or Clec16a-specific shRNA expressing Min6 β-cells incubated with/without 500μM tiron for 48hrs and treated with/without cytokines for the final 24hrs. n=3/group; * $p<0.05$ by two-tailed t-test. (D) qRT-PCR of selected NFκB transcriptional targets (normalized to Hprt) from RNA isolated from shNT or shClec16a-expressing Min6 β-cells in the presence/absence of cytokines for 24hrs. n=3/group. (E) Cellular labile iron pools (LIP; measured in arbitrary units) in shNT or shClec16a-expressing Min6 β-cells treated in the presence/absence of cytokines for 24hrs. n=10/group. *$p<0.05$ by ANOVA. (F) NOS2 mRNA expression (normalized to Cyclophilin A; CYPA) by qRT-PCR from RNA isolated from human islets treated with/without 10 μM lenalidomide for 48hrs and then treated with/without cytokines for the final 24hrs. n=4/group. (G) qRT-PCR of other selected NFκB transcriptional targets (normalized to CYPA) from RNA isolated from human islets treated with/without 10 μM lenalidomide for 48hrs and then exposed to cytokines or vehicle for the final 24hrs. n=4/group.

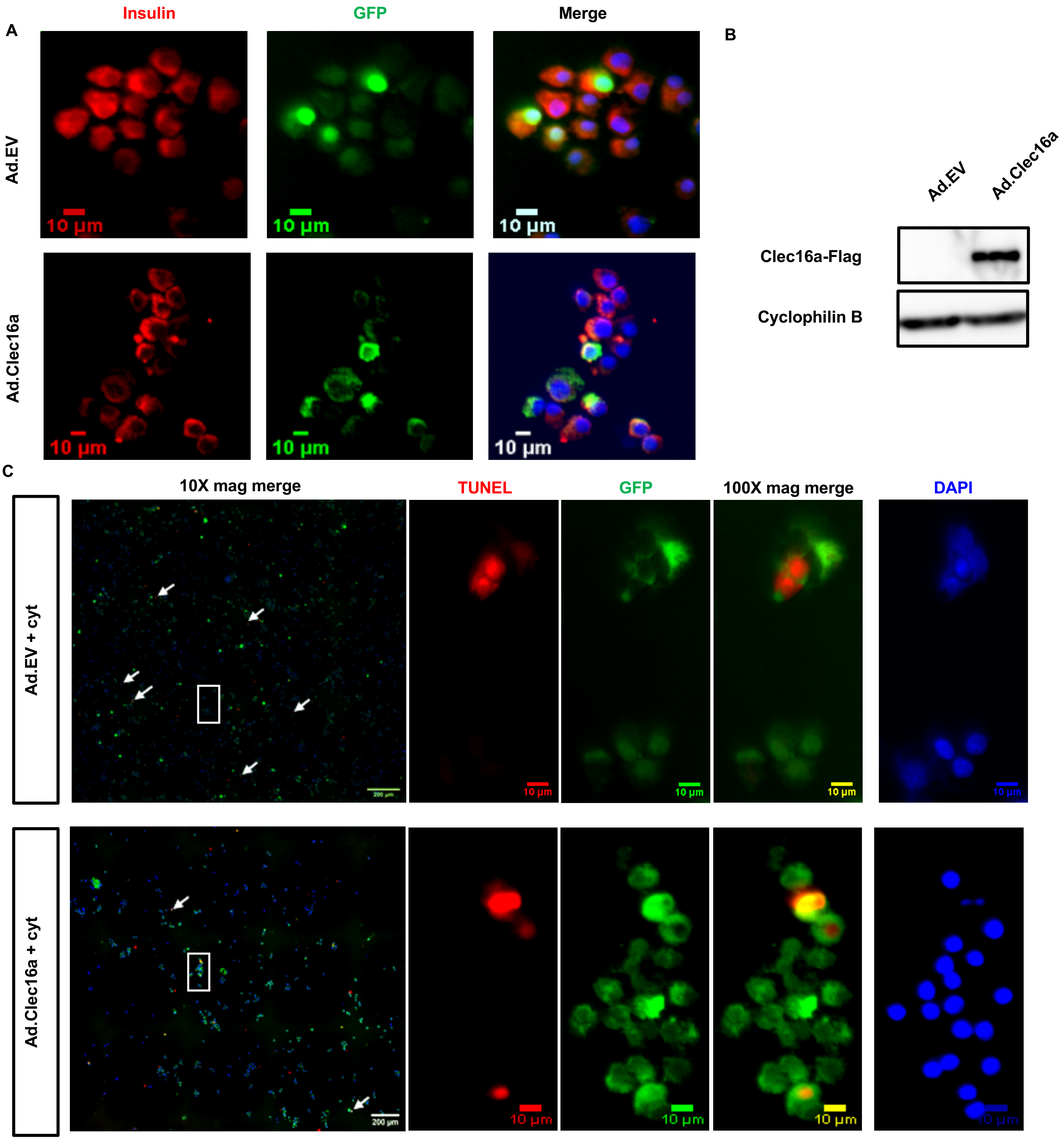

Figure S8. Clec16a overexpression protects against cytokine toxicity in human islets.
(A) Immunofluorescence image at 40X magnification of human islets transduced with empty vector control (Ad.EV) or Clec16a-overexpressing (Ad.Clec16a) adenoviral particles stained for insulin (red), GFP (green), and DAPI (blue). β-cells were identified by insulin immunostaining, and virally transduced cells were identified by GFP immunostaining. Representative of 4 independent experiments. (B) Flag-Clec16a expression by WB performed in Min6 β-cells, 72hrs after overnight transduction with Ad.GFP or Ad.Clec16a adenoviral particles. Cyclophilin B serves as a loading control. Representative of 3 independent experiments. (C) Immunofluorescence images of human islets (at 10X and 100X magnification), transduced with empty vector control (Ad.EV) or Clec16a-overexpressing (Ad.Clec16a) adenoviral particles, and exposed to cytokines or vehicle for 24hrs stained for TUNEL (red), GFP (green), and DAPI (blue). Transduced cells were identified by GFP immunostaining. Representative of 4 independent experiments.

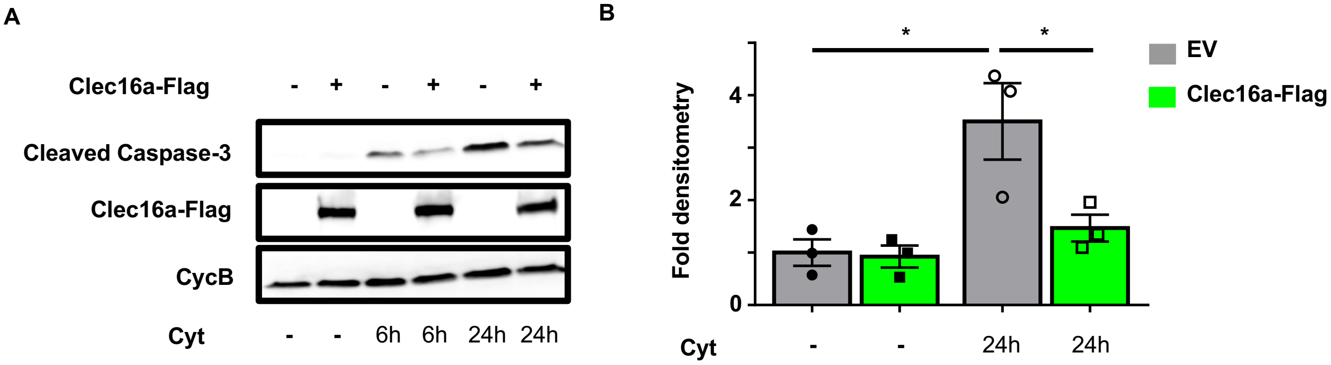

Figure S9. Clec16a overexpression protects against cytokine-mediated apoptosis in Min6 β-cells.
(A) Cleaved caspase 3 and Flag expression by WB in empty vector (EV) or Clec16a-Flag expressing Min6 β-cells treated with cytokines for 6 and 24 hrs. Representative of 3 independent experiments. (B) Cleaved caspase 3 densitometry (normalized to Cyclophilin B) in empty vector (EV) or Clec16a-Flag expressing Min6 β-cells treated with/without cytokines for 24 hrs. n=3/group. *$p<0.05$ by ANOVA.
