# Supplementary material for: Mitophagy protects β cells from inflammatory damage in diabetes
Source: JCI Insight. 2020 Dec 17;5(24):e141138. doi: 10.1172/jci.insight.141138 (PMC7819751; doi:10.1172/jci.insight.141138)
Supplement: Supplemental Table 3 [file jciinsight-5-141138-s093.pdf]

Supplementary Table 3. qRT-PCR primer sequences.

Gene	Target species	Fwd	Rev
Clec16a	Mouse	5’-TGCAGCTGCTACAGACCTTGA-3’	5’-ATACGCCATGATCTCCTCGTC-3’
Nos2	Mouse	5’-ACTGGGACAGCACAGAATGTTCC-3’	5’-CCAAATGTGCTTGTCACCACCAG-3’
Sod2	Mouse	5’-TACAACTCAGGTCGCTCTTCAGC-3’	5’-AGCCTCCAGCAACTCTCCTTT-3’
Fas	Mouse	5’-TTAAAGCTGAGGAGGCGGGTT-3’	5’-CTCAGCCTAGTTTTCAGGTTGGC-3’
Bnip3	Mouse	5’-GCTTGGGGATCTACATTGGAAGG-3’	5’-GTGCAAACACCCAAGGACCAT-3’
Dmt1	Mouse	5’-GCATTGGGTCTGTCTTTCCTG-3’	5’-TGGACACCACTGAGTCAGCAT-3’
Hprt	Mouse	5’-TGCTCGAGATGTCATGAAGGA-3’	5’-CCAGCAGGTCAGCAAAGAACT-3’
NOS2	Human	5’-TGCCCTGGCAATGGAGAGAAA-3’	5’-GCCAAACACAGCGTACCTGAA-3’
SOD2	Human	5’-TAGCTCTTCAGCCTGCACTGA-3’	5’-AGCAACTCCCCTTTGGGTTCT-3’
FAS	Human	5’-TGTCCTCCAGGTGAAAGGAAAGC-3’	5’-TGTACTCCTTCCCTTCTTGGCAG-3’
BNIP3	Human	5’-CTCTGCTGCTCTCTCATTTGCTG-3’	5’-AAAGGTGCTGGTGGAGGTTGT-3’
DMT1	Human	5’-ACCAACGAGCAGGTGGTTGAA-3’	5’-AGTGCAGCAGGCCCAAAGTAA-3’
CYPA	Human	5’-GCGTCTCCTTTGAGCTGTTTGCA-3’	5’-CCACCCTGACACATAAACCCTGGAA-3’
